# Antibiotic resistance genotype, phenotype, and clinical outcomes in patients with Gram-negative infections at Rabin Medical Center in Israel

**DOI:** 10.1128/spectrum.00383-24

**Published:** 2024-11-27

**Authors:** Rachelle E. Koch, Jackson Barth, Andrew E. Clark, Dhara Desai, Jiwoong Kim, Christine A. Pybus, Xiaowei Zhan, Leonard Leibovici, Dafna Yahav, David E. Greenberg

**Affiliations:** 1 Department of Internal Medicine, Tufts Medical Center, Boston, Massachusetts, USA; 2 Department of Statistical Science, Baylor University, Waco, Texas, USA; 3 Department of Pathology, University of Texas Southwestern Medical Center, Dallas, Texas, USA; 4 Department of Internal Medicine, Infectious Diseases and Geographic Medicine, University of Texas Southwestern Medical Center, Dallas, Texas, USA; 5 Quantitative Biomedical Research Center, Department of Clinical Sciences, University of Texas Southwestern Medical Center, Dallas, Texas, USA; 6 Research Authority, Beilinson Hospital, Rabin Medical Center, Petah Tikva, Israel; 7 Sackler School of Medicine, Tel Aviv University, Tel Aviv, Israel; 8 Infectious Diseases Unit, Sheba Medical Center, Ramat Gan, Israel; 9 Department of Microbiology, University of Texas Southwestern Medical Center, Dallas, Texas, USA; Nevada State Public Health Laboratory, Reno, Nevada, USA

**Keywords:** antibiotic resistance, Gram-negative infections, whole-genome sequencing, clinical outcomes

## Abstract

Antibiotic resistance is a major cause of morbidity and mortality. However, a better understanding of the relationship between bacterial genetic markers, phenotypic resistance, and clinical outcomes is needed. We performed whole-genome sequencing on five medically important pathogens (*Acinetobacter baumannii*, *Enterobacter cloacae*, *Escherichia coli*, *Klebsiella pneumoniae*, and *Pseudomonas aeruginosa*) to investigate how resistance genes impact patient outcomes. A total of 168 isolates from 162 patients with Gram-negative infections admitted to Beilinson Hospital at Rabin Medical Center in Israel were included for final analysis. Genomes were analyzed for resistance determinants and correlated with microbiologic and clinical data. Thirty-day mortality from time of culture was 26.5% (43/162). Twenty-nine patients had carbapenem-resistant isolates (29/168, 17.2%), while 63 patients had multidrug-resistant isolates (63/168, 37.5%). Albumin levels were inversely associated with mortality and length of stay, while arrival from a healthcare facility and cancer chemotherapy predicted having a multidrug-resistant isolate. Sequencing revealed possible patient-to-patient transmission events. *bla*_CTX-M-15_ was associated with multidrug-resistance in *E. coli* (OR = 3.888, *P* = 0.023) on multivariate analysis. Increased *bla*_OXA-72_ copy number was associated with carbapenem-resistance in *A. baumannii* (*P* = 0.003) and meropenem minimum inhibitory concentration (*P* = 0.005), yet carbapenem-resistant isolates retained sensitivity to cefiderocol and sulbactam–durlobactam. RJX84154 was associated with multidrug-resistance across all pathogens (*P* = 0.0018) and in *E. coli* (*P* = 0.0024). Low albumin levels were associated with mortality and length of stay in this sample population. *bla*_CTX-M-15_ was correlated with multidrug-resistance in *E. coli*, and *bla*_OXA-72_ depth predicted meropenem minimum inhibitory concentration in *A. baumannii*. RJX84154 may play a role in multidrug-resistance.

**IMPORTANCE:**

While there have been several studies that attempt to find clinical predictors of outcomes in patients hospitalized with bacterial infections, less has been done to combine clinical data with genomic mechanisms of antibiotic resistance. This study focused on a hospitalized patient population in Israel with infections due to medically important bacterial pathogens as a way to build a framework that would unite clinical data with both bacterial antibiotic susceptibility and genomic data. Merging both clinical and genomic data allowed us to find both bacterial and clinical factors that impact certain clinical outcomes. As genome sequencing of bacteria becomes both rapid and commonplace, near real-time monitoring of resistance determinants could help to optimize clinical care and potentially improve outcomes in these patients.

## INTRODUCTION

Antimicrobial resistance is an urgent problem that increases morbidity and mortality (1). In 2017, the World Health Organization outlined the bacterial pathogens that pose the greatest risk to humanity (2, 3). Carbapenem-resistant *Acinetobacter baumannii*, *Pseudomonas aeruginosa*, and Enterobacteriaceae were among the priority bacteria in need of further investigation and alternative therapeutics (4, 5). While molecular epidemiology studies have characterized the worldwide prevalence and distribution of these Gram-negative pathogens, less is known about the intersection of genomic resistance determinants with antimicrobial phenotype and clinical outcomes (6–10). As resistance increases, it will become vital to measure the clinical impact of specific genomic markers and develop diagnostics that allow for a complete view of these myriad resistance mechanisms.

Whole-genome sequencing (WGS) of bacteria represents a high-resolution method for tracking pathogens and broadly characterizing the drivers of antibiotic resistance (11). WGS has enabled phylogenetic tracing of epidemiological trends and correlation of strains with clinical outcomes (12–15). In addition, resistance genes identified through WGS are a predictor of antimicrobial susceptibility patterns (16, 17). WGS has also been used to determine how variation in gene copy number mediates resistance characteristics, which can occur through amplification events or accessory genomes (18–20).

Despite several studies that attempt to determine clinical predictors of outcomes in patients with Gram-negative infections and a growing number of studies that link phenotypic resistance to specific genomic mechanisms, less has been done to merge these types of data together (21–24). In this study, we investigated the relationship between resistance genotype, phenotype, and clinical outcomes in hospitalized patients. We focused on nosocomial infections caused by five high-priority pathogens to better understand the impact of clinical co-morbidities and bacterial resistance genotype on the following microbiological and clinical outcomes: multidrug-resistance, length of stay, and mortality.

## MATERIALS AND METHODS

### Study design and sample collection

This was an observational, prospective cohort study of patients at Beilinson Hospital at Rabin Medical Center in Petah-Tikva, Israel, from May 2019 to June 2020. Clinical data and isolates were obtained systematically by Rabin investigators. The study was approved by the Rabin Research Ethics Committee. The committee waived participants’ consent for this study, in accordance with Israeli legislation. Isolates were sent to UT Southwestern and included within the Texas Infectious Diseases Biorepository (TIDB) under IRB Protocol STU-2018–0319. Each isolate received a unique TIDB number.

One to two bacteremia or respiratory isolates were obtained prospectively from each patient if their infection was caused by the five most common Gram-negative bacteria at Beilinson Hospital: *Acinetobacter baumannii*, *Enterobacter cloacae*, *Escherichia coli*, *Klebsiella pneumoniae*, and *Pseudomonas aeruginosa*. Respiratory isolates were included for analysis once the study physician adjudicated the case as a true infection. Hospital- or ventilator-acquired pneumonia was documented based on criteria described by Horan et al. (25). Patients with polymicrobial infections were not included.

A total of 192 isolates were shipped to UT Southwestern for sequencing. Of the sequenced isolates, 11 isolates without clinical data and 13 without reliable AST data were excluded. In total, 168 isolates from 162 patients were included in the final analysis, as six patients had two isolates each. Patients’ clinical data, including risk factors, such as recent exposure to steroids, cancer chemotherapy, or antimicrobials, were de-identified and extracted from the electronic medical record. The Charlson Comorbidity Index (CCI) was calculated for each patient based on the published criteria (26). Mortality within and outside the hospital was documented by the Ministry of Interior Affairs of Israel. Clinical outcomes included length of stay and 30-day mortality from culture collection (27). Clinical failure (death, fever, or hemodynamic instability on day 5 of appropriate therapy) was not statistically analyzed. Microbiological outcomes included multidrug resistance and carbapenem resistance, which are defined further in a subsequent section.

### Clinical variable definitions

Clinical variables are listed in Table 1 with the following superscript letters for further clarification: (**a**) One isolate was obtained from each patient, except for six patients with two isolates. (**b**) Healthcare facility is defined as admission from a long-term care facility, clinic, or another hospital. (**c**) Blood cancer is defined as past or current leukemia or lymphoma. (**d**) Met. solid tumor refers to metastatic solid tumor. (**e**) CPD refers to chronic pulmonary disease including chronic obstructive pulmonary disease, interstitial lung disease, pulmonary fibrosis, etc. (**f**) Transplant refers to history of solid organ transplant. (**g**) Functionally dependent status is defined as having limited activities of daily living, requiring help with activities of daily living, or being on complete bed rest. (**h**) Healthcare exposures prior to infection are defined as follows: Hospitalization in the 90 days prior to culture collection. Chemotherapy, surgery, steroids, or antibiotics in the 30 days prior to culture collection. (**i**) Antibiotic duration refers to the total number of days the patient was on at least one antibiotic in the 30 days prior to culture collection. Mean and standard deviation exclude patients who took no antibiotics in the 30 days prior to culture collection. (**j**) A pulmonary source of positive culture is defined as a positive bronchoalveolar lavage or sputum culture. (**k**) Time to appropriate empiric therapy at 24 and 48 h is defined as receiving at least one antimicrobial within 1 or 2 days of culture collection, with an isolate susceptible to at least one of the classes of the administered antimicrobials. Not all administered antimicrobials had class-level AST data (*n* = 115 for empiric therapy at 24 h, *n* = 119 for empiric therapy at 48 h). (**l**) Length of stay is defined as number of days from admission to discharge. (**m**) A 30-day mortality is defined as death (all-cause) within 30 days of culture collection. (**n**) Clinical failure is defined as fever or hemodynamic instability within 5 days of culture collection, or death within 30 days of culture collection.

**TABLE 1:** Patient demographic, clinical, and microbiological characteristics^*a*^

	All patients	*Acinetobacter baumannii*	*Enterobacter cloacae*	*Escherichia coli*	*Klebsiella pneumoniae*	*Pseudomonas aeruginosa*
Patient count Isolate count^*a*^	162 168	17 18	6 6	77 78	24 24	38 42
Patient characteristics						
Age, mean (SD), years	69.2 (17.4)	57.7 (21.8)	68.5 (22.7)	75.3 (15.8)	64.9 (15.7)	65.7 (14.4)
Male	78 (48.2)	14 (82.4)	4 (66.7)	30 (39)	9 (37.5)	21 (55.3)
Arrival from						
Healthcare facility^***b***^	26 (15.5)	5 (38.5)	0 (0)	14 (19.7)	3 (15.0)	4 (13.8)
Home	112 (66.7)	8 (61.5)	5 (100)	57 (80.3)	17 (85.0)	25 (86.2)
Comorbidities						
CCI, mean (SD)	2.7 (2.7)	1.6 (1.6)	2.2 (2.8)	3.0 (2.8)	2.2 (2.6)	2.9 (2.9)
MI	45 (26.8)	5 (27.8)	2 (33.3)	19 (24.4)	7 (29.2)	12 (28.6)
CHF	30 (17.9)	3 (16.7)	1 (16.7)	15 (19.2)	3 (12.5)	8 (19.1)
T2DM	42 (25)	2 (11.1)	3 (50)	20 (25.6)	6 (25)	11 (26.1)
Blood cancers^***c***^	15 (8.9)	2 (11.1)	0 (0)	9 (11.5)	1 (4.2)	3 (7.1)
CVD	29 (17.3)	2 (11.1)	2 (33.3)	15 (19.23)	2 (8.3)	8 (19.1)
Met. solid tumor^*d*^	34 (20.2)	1 (5.6)	0 (0)	19 (24.4)	4 (16.7)	10 (23.8)
CPD^***e***^	21 (12.5)	4 (22.2)	2 (33.3)	6 (7.7)	5 (20.8)	4 (9.5)
Transplant^***f***^	18 (10.7)	3 (16.7)	1 (16.7)	5 (6.4)	2 (8.3)	7 (16.7)
Functional capacity dependent^***g***^	79 (54.9)	8 (57.2)	3 (60)	43 (61.6)	8 (38.1)	17 (51.5)
Healthcare exposures prior to infection^***h***^						
Hospitalized	57 (33.9)	8 (44.4)	3 (50)	28 (35.9)	6 (25)	12 (28.6)
Chemotherapy	13 (7.7)	2 (11.1)	0 (0)	6 (7.7)	3 (12.5)	2 (4.8)
Surgery	31 (18.5)	5 (27.8)	1 (16.7)	5 (6.4)	4 (16.7)	16 (38.1)
Steroids	17 (10.1)	4 (23.5)	1 (16.7)	2 (2.6)	3 (12.5)	7 (16.7)
Antibiotic	39 (24.1)	4 (23.5)	1 (16.7)	20 (26.0)	5 (20.8)	9 (23.7)
Antibiotic duration, mean (SD), d^***i***^	7.2 (8.8)	8.5 (4.3)	2.0 (0.0)	3.0 (4.3)	8.6 (8.1)	15.9 (12.3)
Infection characteristics						
Department at date of culture collection						
Medical ward	113 (67.3)	11 (61.1)	3 (50)	66 (84.6)	14 (58.3)	19 (42.2)
Surgical ward	36 (21.4)	2 (11.8)	3 (50)	9 (11.7)	8 (33.3)	14 (33.3)
ICU	19 (11.3)	5 (27.8)	0 (0)	3 (3.9)	2 (8.3)	9 (21.4)
Source of positive culture						
Blood	143 (85.1)	14 (77.8)	6 (100)	78 (100)	24 (100)	21 (50)
Pulmonary^***j***^	25 (14.9)	4 (22.2)	0 (0)	0 (0)	0 (0)	21 (50)
Isolate susceptibility						
Multidrug resistant	63 (37.5)	14 (77.8)	0 (0)	27 (34.6)	12 (50)	10 (23.8)
Carbapenem resistant	29 (17.3)	14 (77.8)	0 (0)	0 (0)	0 (0)	15 (35.7)
Need for ventilator at 48 h	14 (8.3)	7 (38.9)	0 (0)	0 (0)	2 (8.3)	5 (11.9)
Labs on admission						
Creatinine, mean (SD), mg/dL	1.6 (1.3)	1.3 (1.5)	1.7 (1.6)	1.5 (1.1)	1.7 (1.8)	1.7 (1.3)
Albumin, mean (SD), g/dL	3.5 (0.7)	3.3 (0.9)	3.5 (0.6)	3.5 (0.7)	3.4 (0.6)	3.5 (0.7)
CRP, mean (SD), mg/dL	12.3 (10.5)	7.4 (8.1)	8.2 (6.9)	14.1 (10.7)	11.7 (8.9)	11.5 (12.3)
Appropriate empiric therapy at 24 h^***k***^	98 (85.2)	4 (80.0)	6 (100)	57 (87.7)	15 (83.3)	16 (76.2)
Appropriate empiric therapy at 48 h^***k***^	106 (89.1)	4 (66.7)	6 (100)	61 (93.8)	16 (88.9)	19 (79.2)
Outcomes						
Length of stay, mean (SD), d**^*l*^**	20.7 (31.4)	30.9 (29.5)	11.5 (6.6)	9.9 (9.8)	30.9 (61.4)	31.7 (29.9)
30-day mortality^***m***^	43 (26.5)	6 (35.3)	0 (0)	19 (24.7)	7 (29.2)	11 (28.9)
Clinical failure^***n***^	53 (32.7)	7 (41.2)	0 (0)	21 (27.3)	10 (41.7)	15 (39.5)

^*a*^ Data presented as count (%), unless otherwise specified. Percentages for male sex, age, and 30-day mortality are calculated from patient count; all others from isolate count. One isolate was obtained from each patient, except for six patients with two isolates. Abbreviations: CCI, Charlson comorbidity index; MI, myocardial infarction; CHF, congestive heart failure; T2DM, type 2 diabetes mellitus; CVD, cerebrovascular disease; CPD, chronic pulmonary disease; ICU, intensive care unit; CRP, C-reactive protein.

^*b*^ Healthcare facility is defined as admission from a long-term care facility, clinic, or another hospital.

^*c*^ Blood cancer is defined as past or current leukemia or lymphoma.

^*d*^ Met. solid tumor refers to metastatic solid tumor.

^*e*^ CPD refers to chronic pulmonary disease including chronic obstructive pulmonary disease, interstitial lung disease, pulmonary fibrosis, etc.

^*f*^ Transplant refers to history of solid organ transplant.

^*g*^ Functionally dependent status is defined as having limited activities of daily living, requiring help with activities of daily living, or being on complete bed rest.

^*h*^ Healthcare exposures prior to infection are defined as follows: Hospitalization in the 90 days prior to culture collection. Chemotherapy, surgery, steroids, or antibiotics in the 30 days prior to culture collection.

^*i*^ Antibiotic duration refers to the total number of days the patient was on at least one antibiotic in the 30 days prior to culture collection. Mean and standard deviation exclude patients who took no antibiotics in the 30 days prior to culture collection.

^*j*^ A pulmonary source of positive culture is defined as a positive bronchoalveolar lavage or sputum culture.

^*k*^ Time to appropriate empiric therapy at 24 and 48 h is defined as receiving at least one antimicrobial within 1 or 2 days of culture collection, with an isolate susceptible to at least one of the classes of the administered antimicrobials. Not all administered antimicrobials had class-level AST data (n = 115 for empiric therapy at 24 h, n = 119 for empiric therapy at 48 h).

^*l*^ Length of stay is defined as number of days from admission to discharge.

^*m*^ 30-day mortality is defined as death (all-cause) within 30 days of culture collection.

^*n*^ Clinical failure is defined as fever or hemodynamic instability within 5 days of culture collection, or death within 30 days of culture collection.

### Antimicrobial susceptibility testing

Antimicrobial susceptibility testing (AST) was performed at Beilinson Hospital. Isolates were identified using the VITEK 2 system (bioMerieux, Marcy-l’Etoile, France) or MALDI Biotyper System (Bruker Daltonics Inc., Billerica, MA), according to the manufacturer’s instructions. Antimicrobial susceptibility profiles of the isolates were determined by the Etest gradient diffusion method or VITEK 2. Clinical and Laboratory Standards Institute (CLSI) 2020 breakpoints were used (28). Non-susceptibility was defined as intermediate or resistant on AST. Multidrug-resistance (MDR) was defined as non-susceptibility to one or more antimicrobials in three or more distinct drug classes. Carbapenem-resistance (CR) was defined as non-susceptibility to ertapenem, imipenem, or meropenem (28). Differences in antimicrobial categories and intrinsic resistance were accounted for by grouping isolates by species (*Pseudomonas aeruginosa*), genus (*Acinetobacter* spp.), or family (*Enterobacterales*) (29, 30).

For some isolates, additional MIC testing was performed at UT Southwestern for meropenem (Pfizer, NY, USA), cefiderocol (Shionogi, Ltd, Osaka, Japan), and sulbactam–durlobactam (Innoviva Specialty Therapeutics). MIC assays were performed according to CLSI guidelines (28). Iron-depleted cation-adjusted Mueller–Hinton II broth (ID-CAMHB, Thermo Fisher Scientific, Dallas, TX) was used for cefiderocol testing. The MIC plates were read at OD_600nm_ (Cytation5, Biotek, Winooski, VT). For sulbactam–durlobactam, Etest (bioMerieux, Marcy-l’Etoile, France) gradient diffusion was used, with MICs identified from the zone of inhibition. Assays were repeated at least twice, with two technical replicates per assay.

### Whole-genome sequencing

Isolates were received from Rabin Medical Center, and genomic DNA was extracted using the ZymoBIOMICS DNA Miniprep Kit (Zymo Research, Irvine, CA) according to the manufacturer’s protocol. Libraries were prepared using the Nextera DNA Flex Lib Prep Kit (Illumina Inc., San Diego, CA) and sequenced using the Illumina MiSeq platform. Genome assembly and ST determination are described below. Rabin isolates were compared to previously sequenced isolates seen at our center in Texas for genomic comparison purposes. Previously identified antimicrobial resistance genes (ARGs), described in more detail below, were used to confirm the presence, absence, and depth of ARGs within the assembled genomes of the five pathogens (Table S1). The genomes of the Rabin isolates have been deposited into the Sequence Read Archive (SRA; BioProject accession PRJNA1112541) (17).

#### Genome assembly

Trim Galore ( https://www.bioinformatics.babraham.ac.uk/projects/trim_galore/) was used for quality and adapter trimming. SPAdes v.3.14.0 was used for *de novo* genome assembly. The ST was defined by mapping the sequencing reads to the allele sequences from PubMLST (https://pubmlst.org) using Burrows–Wheeler Aligner (BWA) v.0.7.17 (31, 32). For *A. baumannii* and *E. coli*, isolates were labeled using the Institute Pasteur (https://bigsdb.pasteur.fr/) strain taxonomy and nomenclature (33, 34).

#### Antibiotic resistance gene abundance estimation

GeneMarkS-2 was used to predict genes on the genome assembly (35). The protein sequences of all the predicted genes were mapped onto the protein sequences of the antibiotic resistance (AR) genes deposited in the Antibiotic Resistance Genes Database (ARDB) and Comprehensive Antibiotic Resistance Database (CARD) using the BLASTP program of DIAMOND (36–38). The genomic regions of AR genes were defined based on the mapping of protein sequences. The sequencing reads were mapped onto the genome assembly using BWA 0.7.12 (32). The reads mapped to AR gene regions and the unmapped reads were mapped onto the AR protein sequence. The AR protein mapping was converted to mapping of AR gene clusters, and depth of coverage was calculated by a custom script using SAM tools 0.1.19 (39). Mean depth of coverage was calculated for each gene region and normalized by being divided by the following single-copy universal genes: *pgk*, *rplA*, *rplK*, *rplM*, *rplN*, *rplR*, *rpmC*, *rplF*, *rpsM*, *rpsS*, and *rpsH*. These normalized gene depths were used to estimate the copy number of various genes. The differentially abundant genes were identified using statistical significance from the Student’s *t*-test and Wilcoxon rank-sum test implemented in R. A total of 238 ARGs were found to be present in at least one Rabin isolate (Table S1). Resistance genes found to have a depth of coverage greater than 2 were considered to have a high depth of coverage and included for analysis.

#### Genome comparison

MUMmer v.4 was used to compare the genome assemblies. The mutation rate between two genomes was calculated by dividing the number of single-nucleotide polymorphisms (SNPs) by the alignment length, and Jukes–Cantor distance was calculated from the mutation rate. The neighbor-joining tree was generated based on the distances using R packages ape and ggtree (39–41). Core alignments were generated using snippy (https://github.com/tseemann/snippy). The reference genomes of *Pseudomonas aeruginosa* (ASM676v1), *Escherichia coli* (ASM886v2), and *Acinetobacter baumannii* (ASM863263v1) were downloaded from NCBI FTP. RAxML (v8.2.12) and the wrapper in R, ips (https://CRAN.R-project.org/package=ips) with default settings for GTRCAT were used to generate maximum likelihood phylogenetic trees (42, 43).

### Statistics

Univariate and multivariate statistical analyses were performed for four outcomes: 30-day mortality from culture collection, multidrug-resistance, carbapenem-resistance, and length of stay. Aside from length of stay, which was a continuous variable, outcomes were coded as binary variables. *P*-value significance cutoffs were adjusted using the Benjamini–Hochberg procedure. Statistics were performed using R version 4.1.1 (R Core Team 2021) and SAS 9.4 (SAS Institute, Cary NC).

In univariate analyses for mortality, multidrug resistance, and carbapenem resistance, Fisher’s exact test was used for categorical variables, and mean difference *t*-test was used for continuous variables. Adjusted cutoff *P*-values were used: *P* < 0.001 for 30-day mortality *t*-test, *P* < 0.001 for 30-day mortality odds ratio, *P* < 0.001 for MDR *t*-test, *P* < 0.001 for MDR odds ratio, *P* < 0.005 for CR *t*-test, and *P* < 0.001 for CR odds ratio. For multivariate analyses of mortality, multidrug resistance, and carbapenem resistance, logistic regression with stepwise variable selection was performed. Variables were included in the model if *P* < 0.2 in univariate analyses and remained in the model if *P* < 0.3 with the addition of other variables. *bla*_OXA-23_ and *bla*_OXA-72_ were included in multivariate models for *A. baumannii* and *bla*_CTX-M-15_ in multivariate models for *A. baumannii* and *E. coli*. Gene depth values were used in these analyses.

In univariate analyses for length of stay, Spearman correlation was used for continuous variables, and correlation coefficients are shown. Mean difference *t*-test was used for categorical variables, and *P*-values alone are shown. Adjusted cutoff *P*-values were used: *P* < 0.001 for length of stay *t*-test, *P* < 0.01 for length of stay correlation. For multivariate analysis of length of stay, lifetime analysis with backward stepwise variable selection was performed. Correlation estimate is shown for length of stay covariates. All patients who died in-hospital were censored in the multivariate analyses.

## RESULTS

### Demographics, clinical microbiology, and outcomes

Clinical and microbiological data were analyzed from 162 patients (Table 1). Average age was 69.2 ± 17.4 years with nearly even gender distribution. Comorbidities were common in this patient population, and prior myocardial infarction (27%) and type II diabetes mellitus (25%) were frequently observed. Of the patients, 55% required assistance with activities of daily living, and 16% were admitted from another healthcare facility. Of the patients, 34% were hospitalized in the 90 days prior to culture, and 24% received antibiotics in the 30 days prior to culture (average duration: 7.2 ± 8.8 days). Cultures were obtained within 1 day of admission for 53% patients, and within 2 to 7 days of admission for 17% patients.

Regarding clinical course, average length of stay was 20.7 ± 31.4 days (median: 10 days), and 11 patients had a length of stay ≥60 days. Overall, mortality within 30 days of culture collection was 27%, with most pathogens exhibiting similar mortality rates (Table 1, *P* = 0.366). Although not a statistically analyzed outcome, clinical failure rate (which included fever or hemodynamic instability within 5 days of culture collection in addition to 30-day mortality) was 33%.

Antimicrobial resistance was observed in many of these isolates. In total, 38% of isolates were classified as multidrug resistant, and 17% were carbapenem resistant. While MDR was seen in isolates from all pathogens, carbapenem resistance was seen only in *A. baumannii* and *P. aeruginosa*. Carbapenem resistance tracked with all MDR *A. baumannii* isolates; however, this was not the case for *P. aeruginosa*. Ten *P*. *aeruginosa* isolates were both MDR and carbapenem resistant, while an additional five isolates were resistant to only carbapenems.

Of the patients, 85% received appropriate empiric therapy, defined as receiving at least one antimicrobial with *in vitro* activity against the infection isolate within 1 day of culture collection. Those with carbapenem-resistant *Acinetobacter* isolates trended toward greater 30-day mortality compared to carbapenem-susceptible *Acinetobacter* isolates (mortality was observed in 6/15 carbapenem-resistant isolates versus mortality in 0/3 carbapenem-susceptible isolates). In *P. aeruginosa*, those with carbapenem-resistant isolates trended toward greater 30-day mortality than those with carbapenem-susceptible isolates (mortality was observed in 5/15 carbapenem-resistant isolates versus mortality in 6/27 carbapenem-susceptible isolates).

### Various risk factors correlate with antibiotic resistance and clinical outcomes within and across pathogens

**Fig 1:**
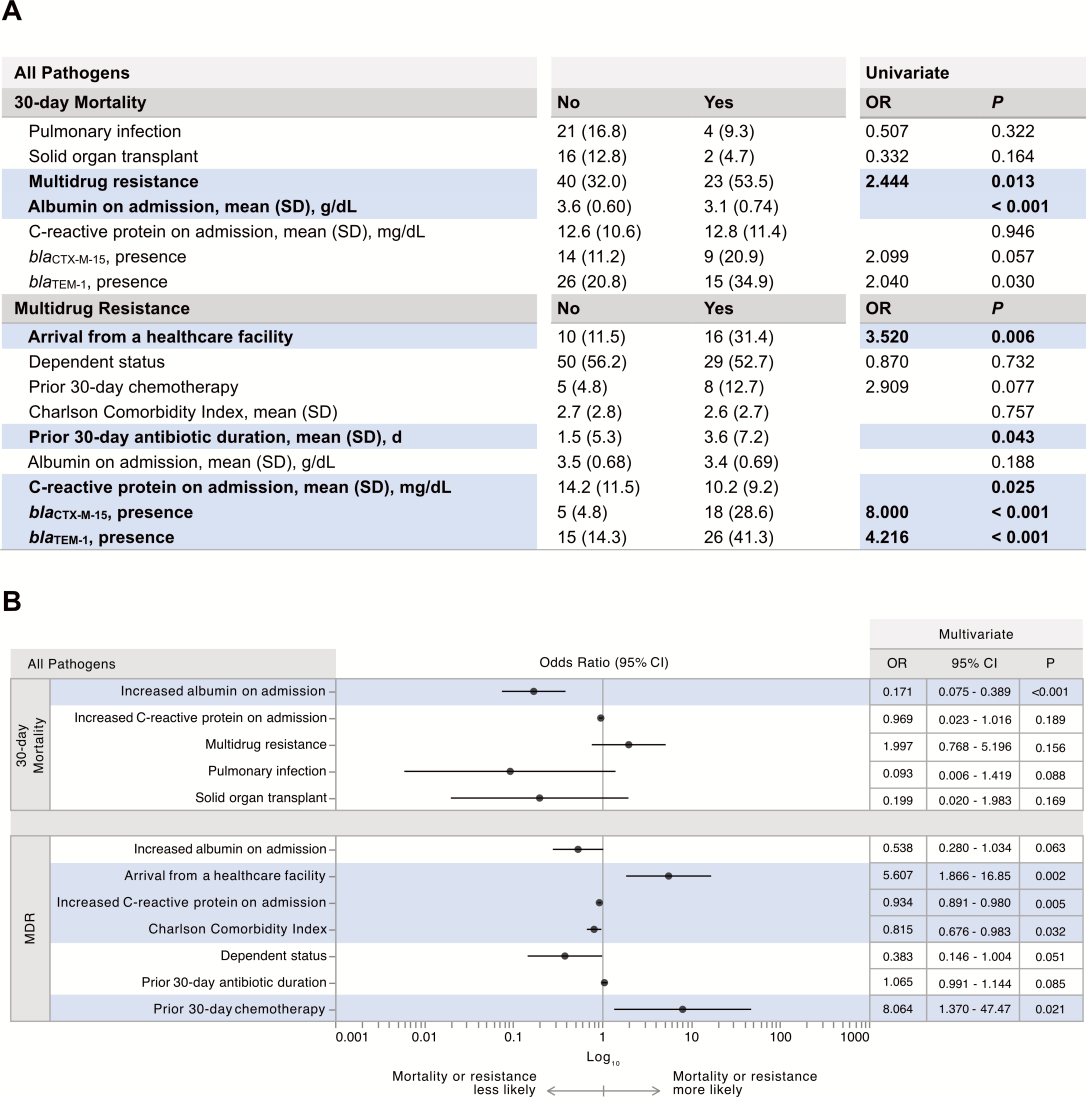
Correlation of risk factors with 30-day mortality or multidrug resistance. (**A**) Univariate analysis across pathogens. Count (%) is reported unless otherwise specified. For the outcome of 30-day mortality, no corresponds to patients who survived, and yes corresponds to patients who died within 30 days of culture collection. For the outcome of multidrug resistance (MDR), no corresponds to patients with non-MDR isolates, and yes corresponds to patients with MDR isolates. (**B**) Multivariate analysis across pathogens. Outcomes are listed vertically on the left. (A and B) OR, odds ratio; *P*, *P*-value; 95% CI, 95% confidence interval. Statistically significant values are bold and highlighted. The univariate significance threshold was adjusted for multiple comparisons, and only univariate associations that were significant or remained in the multivariate model are shown.

Across and within pathogens, multivariate analysis revealed different variables that were associated with the outcomes of having an MDR isolate, 30-day mortality, and length of stay. (Fig. 1**;** Fig. S1; Tables S2 and S3, Supplemental Results). Prior cancer chemotherapy and presentation from a healthcare facility resulted in substantially greater odds of having an MDR infection across all pathogens and in *E. coli* (Fig. 1; Table S3). Low serum C-reactive protein was also associated with MDR infection across all pathogens (Fig. 1). Hypoalbuminemia on culture collection date was associated with increased odds of 30-day mortality across pathogens and in *E. coli*. Prior surgery as well as low albumin and C-reactive protein on admission were associated with increased hospital stays, among other variables (Fig. S1).

### Whole-genome sequencing reveals common circulating sequence types as well as possible transmission events

WGS was performed to better understand the molecular epidemiology of isolates in this patient population and characterize the genomic features of antibiotic resistance. As a geographic comparator group, a previously sequenced collection of isolates from our medical center in Texas was collated with Israeli isolates. We aimed to characterize the extent of shared and unique sequence types (STs) in these specific geographic locations. Most isolates were *E. coli* and *P. aeruginosa* (46% and 25%, respectively). *A. baumannii* had the lowest proportion of unique sequence types (5 STs, 18 isolates), followed by *E. coli* (33 STs, 78 isolates), *P. aeruginosa* (30 STs, 42 isolates), *K. pneumoniae* (22 STs, 24 isolates), and *E. cloacae* (6 STs, six isolates) (Table S1). Here, we will focus on findings related to *P. aeruginosa* isolates.

*P. aeruginosa* isolates from Beilinson Hospital in Israel contained seven of the top 10 globally characterized lineages associated with antibiotic resistance (44). High-risk (multidrug resistant and/or extensively drug resistant) clones ST235, ST111, ST233, ST244, and ST654 were present in isolates from Israel. In contrast, high-risk ST308 and ST357 were present in isolates from Texas. Only three STs were present in both Israel and Texas: ST253, ST309, and ST274. ST data for the four other organisms of interest are discussed further in the Supplemental Results.

**Fig 2:**
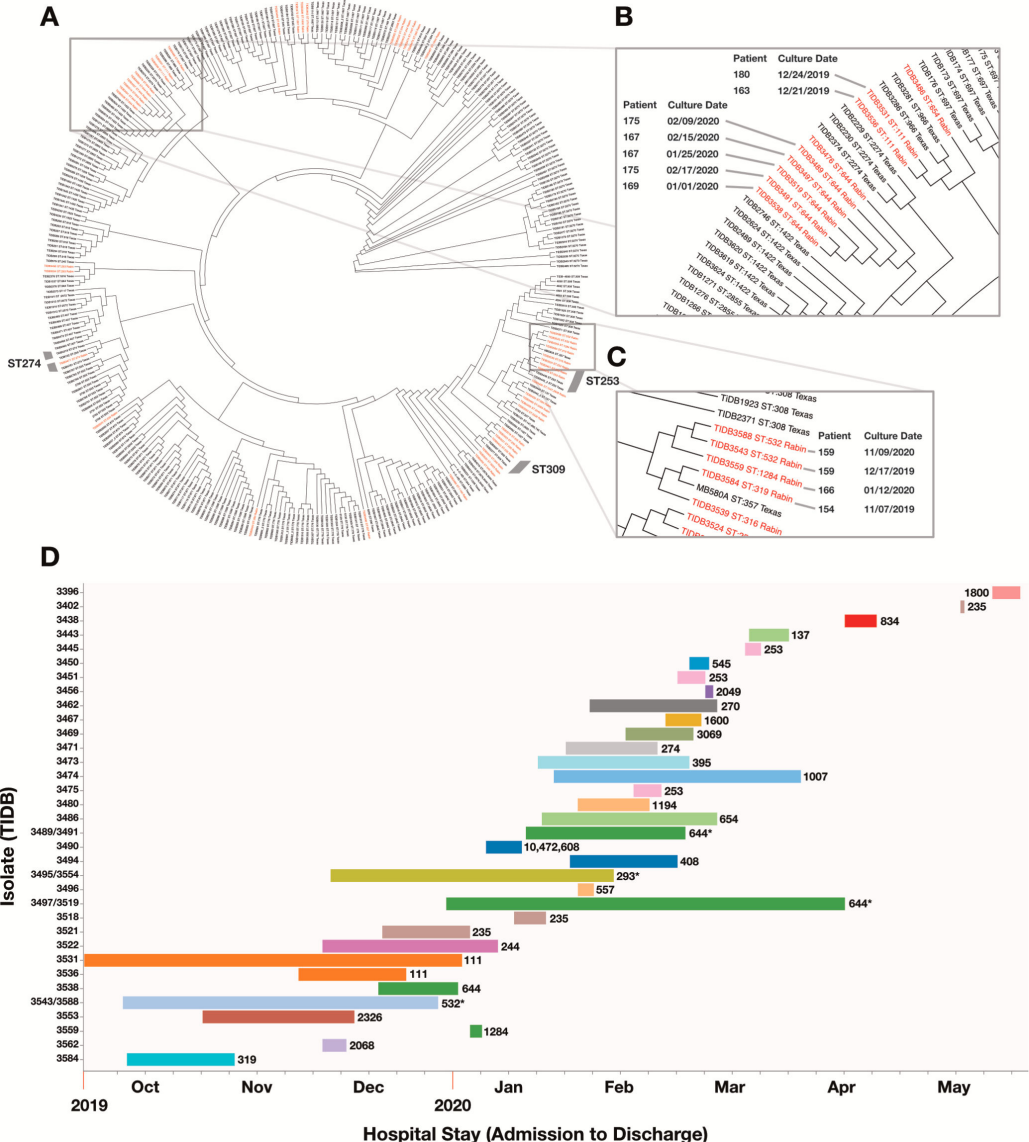
*P. aeruginosa* strains by phylogenetic lineage, location, and hospital stay. (A) *P. aeruginosa* cladogram. Each unique isolate has a unique Texas Infectious Disease Biorepository (TIDB) number. Includes isolates from Rabin Medical Center in Israel (in red) and UT Southwestern Medical Center in Texas (in black). Gray arcs surrounding cladogram indicate sequence types shared between Texas and Israel. (B and C) Various isolates cluster within space and time. (D) *P. aeruginosa* isolate (TIDB) by patient hospital stay. Bars represent hospital stay from admission to discharge for a given isolate and are shown chronologically from October 2019 to May 2020. Sequence type is listed to the right of the respective bar. If two isolates were collected from one patient during a continuous hospital admission, TIDB identifiers were listed as a pair and an asterisk placed beside the sequence type. *Patient 159: ST532, TIDB3543/3588 *Patient 179: ST293, TIDB3495/3554 *Patient 167: ST644, TIDB3497/3519 *Patient 175: ST644, TIDB3489/3491.

Four patients with *P. aeruginosa* infections had two isolates each, and isolate pairs were closely related (Fig. 2A through D). ST644 was the most common *P. aeruginosa* ST, seen in six isolates (clinical data unavailable for TIDB3476) (Fig. 2B). Data from three patients demonstrated possible temporal connections. The hospital stays of Patients 169 and 167 overlapped by 4 days in January 2020, though their culture collection dates differed by 25 days. Pulmonary isolates from Patients 167 and 175 were obtained during continuous hospital admissions for both patients. Their hospital stays overlapped, and all four cultures for both patients were collected within an approximately 3-week span (Fig. 2D). These five isolates showed SNP differences ranging from 108 to 174 depending on the pairwise whole-genome comparison that was made. When analyzing the core genome, the strains were identical with no SNP differences between them. These two comparative genomic analyses indicated that these strains were closely related.

All other *P. aeruginosa* isolates with shared sequence types came from three or fewer patients each. ST111 was present in Patients 163 and 180, whose hospital stays coincided during December 2019; however, their isolates differed by >2,800 SNPs (Fig. 2B). The ST293 and the ST532 isolate pairs both arose from one patient each (Patients 179 and 159, respectively). ST235 was seen in three different patients with no overlap in hospital admission. There were also examples of patients with *A. baumannii* infections with overlapping hospital stays and closely related isolates (Fig. S2, Supplemental Results).

### Beta-lactamase and transposase gene presence and copy number correlate with multidrug-resistance and MIC

**Fig 3:**
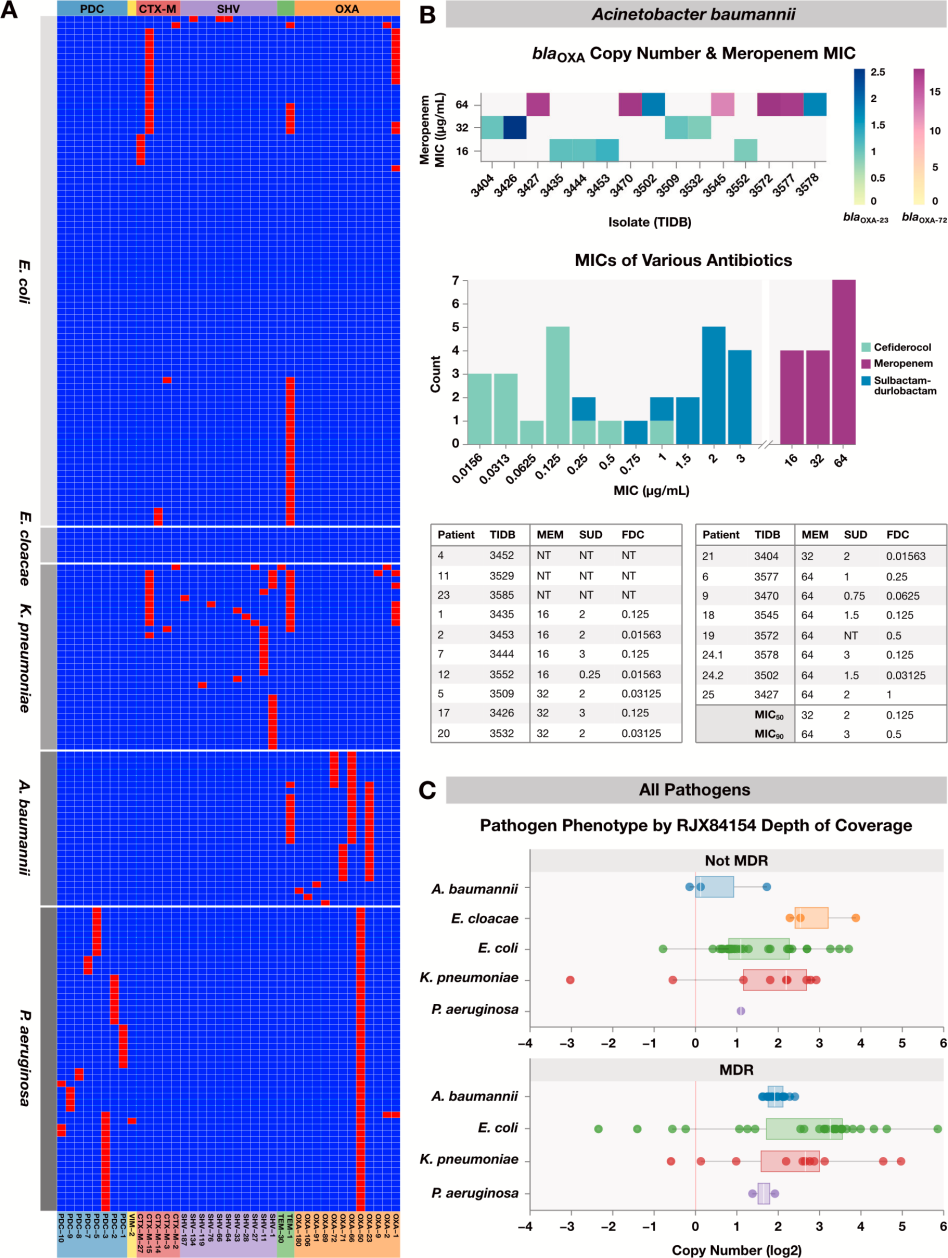
Resistance gene presence and copy number is linked to phenotype. (**A**) Beta-lactamase genes across isolates. Heat map depicts a subset of all resistance genes sequenced. Red for present (at least one copy); blue for absent. (**B**) *bla*_OXA_ family genes are associated with carbapenem resistance in *A. baumannii*, but isolates retain susceptibility to sulbactam–durlobactam and cefiderocol. TIDB numbers identify individual isolates. Fifteen carbapenem-resistant (by AST) *A. baumannii* isolates underwent further phenotypic testing. *bla*_OXA-23_ and *bla*_OXA-72_ were present in isolates with meropenem MICs ranging from 16 to 64 μg/mL. Average copy number for isolates carrying *bla*_OXA-72_ (*n* = 5) was 16.6 ± 2.1. *bla*_OXA-72_ copy number was associated with carbapenem resistance (*P* = 0.003), meropenem MIC (*P* = 0.005), and cefiderocol MIC (*P* = 0.01). Meropenem (MEM) in purple (*n* = 15), sulbactam–durlobactam (SUD) in blue (*n* = 14), cefiderocol (FDC) in teal (*n* = 15). Three isolates from patients 4, 11, and 23 were not multidrug or carbapenem resistant and therefore not tested (NT). (**C**) Depth of coverage of isolates expressing RJX84154 in non-MDR and MDR isolates. RJX84154, an IS26 family transposase, was found in 96/168 (57%) of isolates. Points represent isolates, and isolates without the gene were excluded from the boxplots. No *E. cloacae* isolates were MDR. Mean depth of coverage for isolates that were not MDR was 1.6 ± 2.9 and 7.0 ± 9.3 for those that were MDR. For *E. coli*, mean depth for isolates that were not MDR was 2.0 ± 3.0 and 10.4 ± 11.5 for those that were MDR. Presence of RJX84154 was associated with MDR across all pathogens (*P* = 0.0018) and in *E. coli* (*P* = 0.0024).

Various resistance genes were identified in this data set. *bla*_TEM-1_, *bla*_CTX-M-15_, and *bla*_OXA_ family genes were frequent across isolates in multiple species (Fig. 3A). The average count of beta-lactamase genes per isolate was 1.9 ± 1.3, with a max of six genes in one isolate. *bla*_TEM-1_ was present in a greater proportion of isolates resistant to penicillins and cephalosporins (36.5% of resistant isolates had *bla*_TEM-1_ versus 13.8% of susceptible isolates). In addition, at a pathogen level, *bla*_TEM-1_ was associated with resistance to these antibiotic classes in *A. baumannii*, *E. coli*, *E. cloacae*, and *K. pneumoniae* as a group and remained significant for *E. coli* alone on univariate analysis (*P* < 0.001 for the four pathogens, and *P* = 0.003 for *E. coli*). Both *bla*_TEM-1_ and *bla*_CTX-M-15_ were present in a greater proportion of MDR isolates compared to non-MDR isolates (Fig. 1). Presence of *bla*_CTX-M-15_ was significantly associated with MDR in *E. coli*, and higher average *bla*_CTX-M-15_ copy number was significantly correlated with MDR (Fig. S1).

*bla*_OXA-23_ and *bla*_OXA-72_ were exclusively found in *A. baumannii* (Fig. 3A). Higher average *bla*_OXA-23_ copy number was significantly associated with MDR on univariate analysis (Fig. S1). To determine if presence or copy number of these genes were related to MIC, we performed broth microdilution testing for meropenem in all carbapenem-resistant *A. baumannii* isolates (Fig. 3B). A total of 7/15 isolates had a meropenem MIC of 64 µg/mL. Isolates with a meropenem MIC ≤32 µg/mL all lacked *bla*_OXA-72_. However, the presence of *bla*_OXA-72_ was associated with high-level meropenem resistance (MIC >32 µg/mL). All isolates containing *bla*_OXA-72_ were found to have high copy numbers of this gene, indicating either multiple chromosomal duplications or the presence of plasmid-mediated carriage. Given the high-level carbapenem resistance in these isolates, MICs for cefiderocol and sulbactam–durlobactam were determined. All isolates retained susceptibility to cefiderocol and sulbactam–durlobactam, with MICs ≤ 1 or ≤3 µg/mL, respectively (1, 28).

One *P. aeruginosa* isolate (TIDB3531; ST111) contained VIM-2 metallo-beta-lactamase, which has been implicated in MDR/XDR clones (44). Various PDC genes were distributed across both carbapenem-susceptible and -resistant *P. aeruginosa* isolates. All carbapenem-resistant and carbapenem-intermediate *P. aeruginosa* isolates remained sensitive to cefiderocol, with an MIC range ≤0.03125 to 0.5 µg/mL (Table S1).

RJX84154, an IS*26* family transposase, was found in more than half of all isolates (Fig. 3C) (45). RJX84154 was present in copy numbers of 3.4 to 5.7 across all pathogens except *P. aeruginosa*. *E. coli* and *K. pneumoniae* each had one isolate with very high depth of coverage, at 58.5 in *E. coli* and 31.6 in *K. pneumoniae*. Mean depth of coverage of this transposase for isolates that were not MDR was lower compared to those that were MDR. Presence of RJX84154 was significantly associated with MDR across all pathogens and in *E. coli*. Correlation analysis found certain resistance genes at similar depths to RJX84154 including *dfrA1*, *mrx*, *mph*, *mphR*, *tetR,* and *aadA* (Pearson correlation coefficient 0.628–0.754).

## DISCUSSION

There are numerous studies that assess clinical predictors of outcomes for hospitalized patients with Gram-negative infections, and there are many studies that correlate antibiotic phenotypic resistance to specific genomic markers. However, few have attempted to build frameworks that merge these large independent data sets together. In this study, we assessed the relationship between clinical and bacterial data in hospitalized patients with Gram-negative infections at a large hospital in Israel. After characterizing the impact of patient risk factors on phenotypic and clinical outcomes, we examined pathogen sequence type and compared it to hospital admission data to identify potential transmission events. We then aimed to understand how antibiotic resistance genes predicted phenotype and outcomes from a few different perspectives. We investigated how gene presence and copy number related to multidrug-resistance and minimum inhibitory concentration for certain antibiotics. In this data set, antibiotic resistance genes were not found to predict length of stay or 30-day mortality. Although the data set was small from a specific pathogen point of view, it presented important observations.

Overall, the study population was found to have a moderate number of comorbidities, and over a quarter of the population died within 30 days of culture. MDR and carbapenem resistance was frequently observed in all pathogens except *Enterobacter*, which was limited by small sample size. A variety of exposures in the 30–90 days preceding culture collection, including hospitalization, cancer chemotherapy, surgery, and steroid or antibiotic use, impacted outcomes across pathogens or in specific pathogens. Additionally, admission from another healthcare facility was associated with having an MDR pathogen, a previously documented association. In one recent study, the percentage of antimicrobial resistant isolates in long-term care facilities in Europe was nearly equivalent to that of acute care hospitals (46).

Two biomarkers, albumin and C-reactive protein, were associated with mortality and having an MDR isolate across all pathogens, respectively. Other studies have found low albumin to be associated with poor outcomes in various infections including bacteremia (47–49). C-reactive protein has been shown to distinguish infection from colonization in *A. baumannii* and predict death from this and other pathogens (50, 51). Nevertheless, it is unclear whether C-reactive protein predicts infection with MDR versus non-MDR pathogens. C-reactive protein may indicate differences in fitness among these groups of pathogens, but further studies are needed to characterize this relationship.

At the pathogen level, duration of prior antibiotic exposure was associated with being carbapenem-resistant in *Pseudomonas* isolates. One study in adult patients showed links between prior antibiotic use and carbapenem-resistant *Pseudomonas aeruginosa* (CRPA) infections (52). Importantly, 100% of carbapenem-resistant isolates were sensitive to cefiderocol (*A. baumannii* and *P. aeruginosa*) and sulbactam–durlobactam in the case of *A. baumannii*. Cefiderocol demonstrates activity in both pathogens, while sulbactam–durlobactam is active against *A. baumannii* and was recently approved for use (53–58). These antibiotics could be considered in patients who are at high risk for or have infections resistant to carbapenems.

Whole-genome sequencing allowed us to explore the molecular epidemiology of these isolates and identify the genomic determinants of resistance that were circulating in this hospitalized population. We compared the sequence types of isolates to each other, though use of an external reference genome is a valid method of comparison and lineage analysis (42). ST analysis and SNP level analysis (both whole- and core-genome analysis) demonstrated that possible transmission events occurred among some clusters of patients with *A. baumannii* and *P. aeruginosa* isolates. It is unknown whether these events were from a common environmental source or through patient-to-patient spread, although both have been described (59–63). Genomics could provide near real-time tracking of MDR pathogen spread at both the hospital and community level.

Whole-genome sequencing also enables characterization of antibiotic resistance genes. Presence of certain resistance genes was found to predict phenotypic resistance in specific pathogens. In *E. coli*, *bla*_TEM-1_ predicted beta-lactam resistance, and *bla*_CTX-M-15_ presence and copy number were associated with MDR in multivariate analysis. These genes have been found in other surveys of resistant pathogens (64). For narrow spectrum beta-lactamases, such as *bla*_TEM-1_, copy number was shown to impact the activity of piperacillin–tazobactam (65). We have demonstrated similar findings for *bla*_OXA-1_ and *bla*_CTX-M-15_ (18).

Gene depth of coverage correlates with resistance as well as minimum inhibitory concentration. *bla*_OXA-23_ and *bla*_OXA-72_ were present in carbapenem-resistant *A. baumannii* isolates and have been reported as resistance determinants for this antibiotic class (66). Additional testing demonstrated that *bla*_OXA-23_ had two- to fourfold lower meropenem MICs than *bla*_OXA-72_ containing strains, though *bla*_OXA-72_ was found at higher gene depth than *bla*_OXA-23_. It is unclear whether the MIC differences were related to high copy numbers of *bla*_OXA-72_ relative to *bla*_OXA-23_, whether there are intrinsic properties that differentiate activity of these two carbapenemases, or whether the variation in MIC is simply within testing error. Copy number and MIC may be positively correlated, but sample size limited further exploration of this hypothesis.

Though not a beta-lactamase, RJX84154, an IS*26*-like transposase described 5 years ago in *E. coli*, was seen in high copy number in *E. coli* and *K. pneumoniae* (45). Since it was correlated with MDR across pathogens and in *E. coli,* it is possible that this element carried at least one resistance gene with it. Various publications have described several resistance genes as being carried within IS*26*-like elements on plasmids. This includes genes conferring beta-lactam resistance, aminoglycoside resistance, and macrolide resistance (67–69). In addition, IS*26*-mediated amplification of beta-lactamase genes has been found to induce a resistance phenotype (18). In *Klebsiella*, progression from colonization to infection has been associated with infection-associated genes on plasmids, which are driven in part by certain transposases (70). Indeed, we found resistance genes, as previously identified in these studies, that were at a similar depth of coverage as RJX84154. While our sequencing efforts were based on short-read data only, future studies will better characterize what resistance genes may be associated with the RJX84154 transposase in these isolates.

Overall, antibiotic resistance genes were seen in low frequency across isolates in our data set: 75% of individual resistance genes were present in no more than six isolates of the 168 isolates analyzed. As such, it was difficult to draw conclusions as to their individual impact on outcomes. Neither presence nor copy number of antibiotic resistance genes were found to predict length of stay or 30-day mortality in this data set. It is possible that this negative finding is a product of low gene frequency. Although not studied here, the total number of beta-lactamase genes per isolate (overall gene burden) should be investigated as a potential predictor of patient survival. Grouping beta-lactamase genes by family or interrogating other non-beta-lactamase resistance genes may also help predict clinical outcomes.

Limitations included the retrospective design, small sample size, and some ambiguous or missing data. As cause of death was not clearly recorded, 30-day mortality from the time of culture was used as an approximation of deaths due to infection. It is possible that mortality within the 30-day window was unrelated to the infection studied. We limited our analyses to mainly beta-lactamase genes, but it is likely that other resistance genes are driving certain phenotypes or clinical outcomes.

Future studies will further elucidate the relationship between pathogen genomic data and clinical outcomes using a larger sample size. It would be worthwhile to clarify the relationship between resistance gene presence and copy number on resistance phenotype. A better understanding of the drivers of phenotypic resistance and survival in these patients will allow for innovation in diagnostic modalities and treatment interventions in the future.

### Key points

In hospitalized patients with infections caused by Gram-negative bacteria, 30-day mortality was high. Low albumin levels were associated with 30-day mortality and length of stay. *bla*_CTX-M-15_ and an IS*26*-like transposase, RJX84154, were associated with an MDR phenotype depending on the pathogen. *bla*_OXA-72_ copy number was associated with increased meropenem MIC in *A. baumannii*. Resistance genes did not correlate with clinical outcomes.

## Data Availability

The genomes of the Rabin isolates have been deposited into the Sequence Read Archive (SRA) under BioProject accession PRJNA1112541.

## SUPPLEMENTAL MATERIAL

Supplemental material spectrum.00383-24-s0001.docx Supplemental results, tables, and figures.

Table S1 spectrum.00383-24-s0002.pdf AST and antibiotic resistance genes present in Rabin isolates.

ASM does not own the copyrights to Supplemental Material that may be linked to, or accessed through, an article. The authors have granted ASM a non-exclusive, world-wide license to publish the Supplemental Material files. Please contact the corresponding author directly for reuse.
