# Supplementary material for: Antibiotic resistance genotype, phenotype, and clinical outcomes in patients with Gram-negative infections at Rabin Medical Center in Israel
Source: Microbiol Spectr. 2024 Nov 27;13(1):e00383-24. doi: 10.1128/spectrum.00383-24 (PMC11705905; doi:10.1128/spectrum.00383-24)
Supplement: Supplemental material — Supplemental results, tables, and figures. [file spectrum.00383-24-s0001.docx]

**Supplemental Material**

**Supplemental Results**

For *E. coli* isolates, low albumin and surgery in the month preceding culture collection date were correlated with 30-day morality, while cognitive impairment was associated with increased odds of death in *P.* *aeruginosa* (**Supplemental Figure 1**). In *P. aeruginosa* significant risk factors for carbapenem-resistance were found, including duration of antibiotic use and steroid use (7-fold increase in risk) in the month preceding culture (**Supplemental Figure 1**).

Prior surgery, hospitalization, dependent functional status, and increased prior antibiotic count were significantly correlated with length of stay in *E. coli* isolates. Interestingly, admission from a healthcare facility was associated with decreased length of stay in *E. coli* isolates. Low albumin was significantly correlated with increased length of stay in *K. pneumoniae* isolates. Bloodstream infection and a higher Charlson score were significantly associated with increased length of stay in *P. aeruginosa* isolates. Prior hospitalization was correlated with decreased length of stay in *P. aeruginosa* isolates. (**Supplemental Table 3**).

**Whole-genome sequencing reveals common circulating sequence types as well as possible transmission events.**

The complete list of AST data and resistance genes that were present or absent for the Rabin isolates are contained in **Supplemental Table 1**. For comparison purposes, isolates obtained from the Clements University Hospital in Dallas, TX that had been genome sequenced were used to compare the prevalence of STs among these pathogens. Of the six *E. cloacae* sequence types, none were ST114, which has been detected internationally. [1] In *K. pneumoniae,* ST23 and ST45 were observed in two patients each, though it does not appear that either of these sequence types were shared through a hospital transmission event. ST17 and ST307 were found in one *K. pneumoniae* isolate each. ST17 is an ESBL-carrying clone, and ST307 is a globally distributed high-risk sequence type. [2, 3]

ST2 and ST3 were the most common sequence types in *A. baumannii* (**Supplemental Figure 2**). ST2 and ST3 were found in isolates from both Israel and Texas. ST2 is globally disseminated and has been characterized in the United States, Israel, Italy, Greece, Jordan, Egypt, and New Zealand between 2010-2018. [4-7] ST3 has been observed in Israel recently as well. [8] All but two ST2 isolates were obtained from unique patients. Patient 24 presented with bacteremia on two separate hospital admissions, with both isolates identified as ST2. These two isolates were closely related with 110 SNPs differing between them (5 SNPs at the core genome level).

Patients 2 (TIDB3453), 7 (TIDB3444), and 17 (TIDB3426) presented with ST3 *A. baumannii* and exhibited overlapping hospital stays from mid-February to mid-April 2020, with blood culture collection dates of March 12, March 19, and April 3, respectively. Whole genome comparative analysis revealed a 65-82 SNP difference between these three isolates, and core genome analysis revealed a 25-127 SNP difference between the isolates. Genomic analyses therefore suggest that these three isolates were closely related. Whole genome comparison of other ST3 isolates demonstrated greater SNP level differences, ranging from 179-1095 SNPs between isolate comparisons.

The most common sequence type in *E. coli* was ST43 (ST131), which is globally disseminated, and was observed in both Israel and Texas [9, 10]. ST3 was the second most common sequence type but only seen in Israel. ST43 was seen throughout the study data collection timespan. In December 2020, five patients were hospitalized and found to have isolates positive for ST3 (TIDB3547, 3555, 3563, 3565, 3568) and all isolates cluster based phylogenetic analysis; however, the number of SNPs between sequence type ranged from 327-11,535 depending on the sequence-to-sequence comparison. A similar clustering pattern was seen with a different set of five patients, who were hospitalized mid-April to mid-May 2020 with isolates identified as ST1 (TIDB3398, 3409, 3416, 3428, 3516) and these isolates also had a number of SNPs that differed between them (4330-9546 SNPs difference).

Other patients with overlapping hospital admissions also revealed isolates with identical *E. coli* sequence types. Three patients were seen with ST4 (TIDB3499, 3487, 3483) and ST478 (TIDB3437, 3436, 3410), and two patients were seen with ST6 (TIDB3441, 3447) and ST52 (TIDB3424, 3425). Patient 43 was the only patient who had two *E. coli* isolates (TIDB3484, 3487). The isolates were two unique sequence types, ST972 and ST4, even though they derived from cultures taken within a week of each other from this patient. Despite having a number of patients with similar ST that were found during distinct periods of time, the *E. coli* isolates in this study seemed to be genetically diverse.

**Supplemental Table 1. AST and Antibiotic Resistance Genes Present in Rabin Isolates**

See provided Excel file.

**Supplemental Table 2. Correlation of risk factors with length of stay.**

| All Pathogens |  | Univariate | | Multivariate | | |
| --- | --- | --- | --- | --- | --- | --- |
| Variable | **Length of Stay** | **CC** | ***P*** | **CE** | **95% CI** | ***P*** |
| Male |  |  | 0.323 | **-0.404** | **-0.783 – (-0.025)** | **0.037** |
| No | 23.0 (23.0) |  |  |  |  |  |
| Yes | 18.5 (37.4) |  |  |  |  |  |
| Arrival from a healthcare facility |  |  | 0.228 | **-0.548** | **-1.015 – (0.080)** | **0.022** |
| No | 20.2 (34.5) |  |  |  |  |  |
| Yes | 14.4 (17.9) |  |  |  |  |  |
| Need for a ventilator at 48 hours |  |  | 0.058 | **0.879** | **0.154 – 1.604** | **0.018** |
| No | 19.0 (30.8) |  |  |  |  |  |
| Yes | 38.7 (33.0) |  |  |  |  |  |
| Solid organ transplant |  |  | 0.344 | **-1.344** | **-1.926 – (-0.761)** | **< 0.001** |
| No | 21.3 (32.5) |  |  |  |  |  |
| Yes | 15.7 (21.6) |  |  |  |  |  |
| Prior 30-day surgery |  |  | **0.022** | **0.882** | **0.370 – 1.394** | **< 0.001** |
| No | 18.0 (31.9) |  |  |  |  |  |
| Yes | 31.3 (27.2) |  |  |  |  |  |
| Age |  | **-0.337** | **< 0.001** | **-0.023** | **-0.034 – (-0.011)** | **< 0.001** |
| Charlson Comorbidity Index |  | -0.027 | 0.744 | -0.056 | -0.127 – 0.016 | 0.127 |
| Albumin on admission |  | 0.016 | 0.844 | **-0.570** | **-0.877 – (-0.264)** | **< 0.001** |
| C-reactive protein on admission |  | -0.135 | 0.107 | **-0.022** | **-0.040 – (-0.004)** | **0.015** |

CC, correlation coefficient; CE, correlation estimate; *P*, *P*-value; 95% CI, 95% confidence interval. Length of stay is defined as number of days from admission to discharge. For categorical variables, mean length of stay is listed in number of days and standard deviation in parentheses. For continuous variables, correlation coefficient is listed. Statistically significant values are bold and highlighted. The univariate significance threshold was adjusted for multiple comparisons, and only univariate associations that were significant or remained in the multivariate model are shown.

**Supplemental Table 3. Correlation of risk factors with length of stay, by pathogen.**

| *Escherichia coli* |  | Univariate | | Multivariate | | |
| --- | --- | --- | --- | --- | --- | --- |
| Variable | **Length of Stay** | **CC** | ***P*** | **CE** | **95% CI** | ***P*** |
| Male |  |  | 0.119 | **-0.589** | **-0.993 – (-0.185)** | **0.004** |
| No | 14.0 (12.7) |  |  |  |  |  |
| Yes | 7.2 (6.2) |  |  |  |  |  |
| Arrival from a healthcare facility |  |  |  | **-0.557** | **-1.007 – (-0.106)** | **0.015** |
| No | 10.7 (10.6) |  |  |  |  |  |
| Yes | 6.7 (4.6) |  |  |  |  |  |
| Dependent status |  |  |  | **0.629** | **0.258 – 1.000** | **< 0.001** |
| No | 9.5 (11.4) |  |  |  |  |  |
| Yes | 10.2 (8.8) |  |  |  |  |  |
| Prior 90-day hospitalization |  |  | 0.086 | **0.500** | **0.098 – 0.902** | **0.015** |
| No | 8.1 (7.8) |  |  |  |  |  |
| Yes | 12.6 (12.0) |  |  |  |  |  |
| Prior 30-day surgery |  |  | 0.405 | 0.706 | -0.031 – 1.442 | 0.060 |
| No | 9.6 (9.8) |  |  |  |  |  |
| Yes | 14.2 (11.0) |  |  |  |  |  |
| CCI |  | 0.155 | 0.196 | -0.064 | -0.140 – 0.011 | 0.094 |
| Prior 30-day antibiotic count |  | **0.506** | **< 0.001** |  |  |  |
| Prior 30-day antibiotic duration |  | **0.552** | **< 0.001** | **0.324** | **0.064 – 0.585** | **0.015** |
|  |  |  |  |  |  |  |
| *Klebsiella pneumoniae* |  | **Univariate** | | **Multivariate** | | |
| Variable | **Length of Stay** | **CC** | ***P*** | **CE** | **95% CI** | ***P*** |
| Male |  |  | 0.147 | 0.471 | -0.290 – 1.232 | 0.223 |
| No | 11.0 (8.3) |  |  |  |  |  |
| Yes | 42.3 (75.3) |  |  |  |  |  |
| Albumin on admission |  | **-0.600** | **0.003** | **-1.470** | **-2.027 – (-0.914)** | **< 0.001** |
|  |  |  |  |  |  |  |
| *Pseudomonas aeruginosa* |  | **Univariate** | | **Multivariate** | | |
| Variable | **Length of Stay** | **CC** | ***P*** | **CE** | **95% CI** | ***P*** |
| Prior 90-day hospitalization |  |  | **< 0.001** | **-1.381** | **-2.214 – (-0.548)** | **0.001** |
| No | 41.2 (31.2) |  |  |  |  |  |
| Yes | 11.2 (11.0) |  |  |  |  |  |
| Prior 30-day chemotherapy |  |  | **0.001** |  |  |  |
| No | 33.2 (30.0) |  |  |  |  |  |
| Yes | 4.5 (4.9) |  |  |  |  |  |
| Pulmonary isolate |  |  | 0.159 | **-0.988** | **-1.825 – (-0.150)** | **0.021** |
| No | 24.8 (26.2) |  |  |  |  |  |
| Yes | 38.6 (32.5) |  |  |  |  |  |
| Charlson Comorbidity Index |  |  |  | **0.153** | **0.008 – 0.298** | **0.039** |
| Prior 30-day antibiotic count |  | **0.491** | **0.002** |  |  |  |
| Prior 30-day antibiotic duration |  | **0.585** | **< 0.001** |  |  |  |
| Creatinine on admission |  | **0.467** | **0.004** |  |  |  |

CC, correlation coefficient; CE, correlation estimate; *P*, *P*-value; 95% CI, 95% confidence interval. For categorical variables, mean length of stay is listed in number of days and standard deviation in parentheses. For continuous variables, correlation coefficient is listed. Statistically significant values are bold and highlighted. The univariate significance threshold was adjusted for multiple comparisons, and only univariate associations that were significant or remained in the multivariate model are shown.

**Supplemental Figure 1. Correlation of risk factors with 30-day mortality and multidrug resistance, by pathogen.**

**A**

| *Acinetobacter baumannii* |  |  | Univariate | |
| --- | --- | --- | --- | --- |
| 30-day Mortality | **No** | **Yes** | **OR** | ***P*** |
| Age, mean (SD), y | 53.3 (19.8) | 66.7 (24.8) | 1.833 | 0.662 |
| Multidrug Resistance | **No** | **Yes** | **OR** | ***P*** |
| *bla*_OXA-23_, mean (SD), depth | 0 (0) | 0.96 (0.77) |  | **< 0.001** |

| *Klebsiella pneumoniae* |  |  | Univariate | |
| --- | --- | --- | --- | --- |
| 30-day Mortality | **No** | **Yes** | **OR** | ***P*** |
| Dependent status | 4 (23.5) | 4 (57.1) | 5.500 | 0.103 |
| Age, mean (SD), y | 61.4 (16.5) | 73.4 (9.9) |  | 0.032 |
| Albumin on admission, mean (SD), g/dL | 3.4 (0.68) | 3.2 (0.39) |  | 0.273 |
| Multidrug Resistance | **No** | **Yes** | **OR** | ***P*** |
| *bla*_CTX-M-15_, mean (SD), depth | 0 (0) | 1.15 (1.16) |  | **0.006** |

| *Pseudomonas aeruginosa* |  |  | Univariate | |
| --- | --- | --- | --- | --- |
| 30-day Mortality | **No** | **Yes** | **OR** | ***P*** |
| Cognitive impairment | 3 (9.7) | 4 (36.4) | 7.000 | 0.046 |
| Age, mean (SD), y | 66.0 (14.2) | 64.9 (15.7) |  | 0.837 |
| Charlson Comorbidity Index, mean (SD) | 2.5 (3.0) | 3.8 (2.6) |  | 0.197 |
| Prior 30-day antibiotic count, mean (SD) | 1.9 (3.5) | 0.91 (2.7) |  | 0.055 |
| Multidrug Resistance | **No** | **Yes** | **OR** | ***P*** |
| Prior 90-day hospitalization | 11 (34.4) | 1 (10.0) | 0.212 | 0.165 |
| Age, mean (SD), y | 68.7 (13.7) | 56.2 (13.1) |  | 0.019 |
| Carbapenem Resistance | **No** | **Yes** | **OR** | ***P*** |
| Need for ventilator at 48 hours | 2 (7.4) | 3 (20.0) | 3.125 | 0.244 |
| Cognitive impairment | 5 (18.5) | 2 (13.3) | 0.677 | 0.667 |
| Prior 90-day hospitalization | 8 (29.6) | 4 (26.7) | 0.864 | 0.839 |
| Prior 30-day steroids | 2 (7.4) | 5 (33.3) | 6.250 | 0.046 |
| Age, mean (SD), y | 70.4 (13.3) | 57.2 (12.9) |  | **0.004** |
| Prior 30-day antibiotic duration, mean (SD), d | 2.0 (6.3) | 11.1 (12.4) |  | 0.015 |

| *Escherichia coli* |  |  | Univariate | |
| --- | --- | --- | --- | --- |
| 30-day Mortality | **No** | **Yes** | **OR** | ***P*** |
| Prior 30-day chemotherapy | 1 (1.7) | 5 (26.3) | **20.72** | **0.003** |
| Prior 30-day surgery | 3 (5.1) | 2 (10.5) | 2.196 | 0.590 |
| Charlson Comorbidity Index, mean (SD) | 2.6 (2.7) | 3.9 (2.9) |  | 0.107 |
| Prior 30-day antibiotic count, mean (SD) | 0.29 (5.9) | 0.74 (1.4) |  | 0.192 |
| Prior 30-day antibiotic duration, mean (SD), d | 0.25 (0.51) | 2.4 (4.8) |  | 0.070 |
| Albumin on admission, mean (SD), g/dL | 3.7 (0.51) | 2.9 (0.72) |  | **< 0.001** |
| Multidrug Resistance | **No** | **Yes** | **OR** | ***P*** |
| Arrival from a healthcare facility | 6 (13.6) | 8 (29.6) | 2.670 | 0.107 |
| Dependent status | 28 (63.6) | 15 (55.6) | 0.720 | 0.499 |
| Prior 90-day hospitalization | 14 (27.5) | 14 (51.9) | 2.846 | 0.035 |
| Prior 30-day chemotherapy | 2 (3.9) | 4 (14.8) | 4.261 | 0.108 |
| Charlson Comorbidity Index, mean (SD) | 2.9 (2.8) | 3.1 (2.9) |  | 0.717 |
| C-reactive protein on admission, mean (SD), mg/dL | 16.1 (10.7) | 11.2 (9.8) |  | 0.057 |
| *bla*_CTX-M-15_, presence | 5 (9.8) | 11 (40.7) | **6.325** | **0.002** |
| *bla*_CTX-M-15_, mean (SD), depth | 0.13 (0.43) | 0.58 (0.91) |  | 0.021 |

**B**

**A) Univariate analysis, by pathogen.** Count (%) is reported unless otherwise specified. For *A. baumannii*, all isolates that were multidrug resistant were also carbapenem resistant. **(B) Multivariate analysis, by pathogen.** Outcomes are listed vertically on the left. Some confidence intervals exceed displayed axes. **(A & B)** OR, odds ratio; *P*, *P*-value; 95% CI, 95% confidence interval. Statistically significant values are bold and highlighted. The univariate significance threshold was adjusted for multiple comparisons, and only univariate associations that were significant or remained in the multivariate model are shown.

**Supplemental Figure 2. Phylogenetic analysis of *Acinetobacter baumannii* isolates.**

**
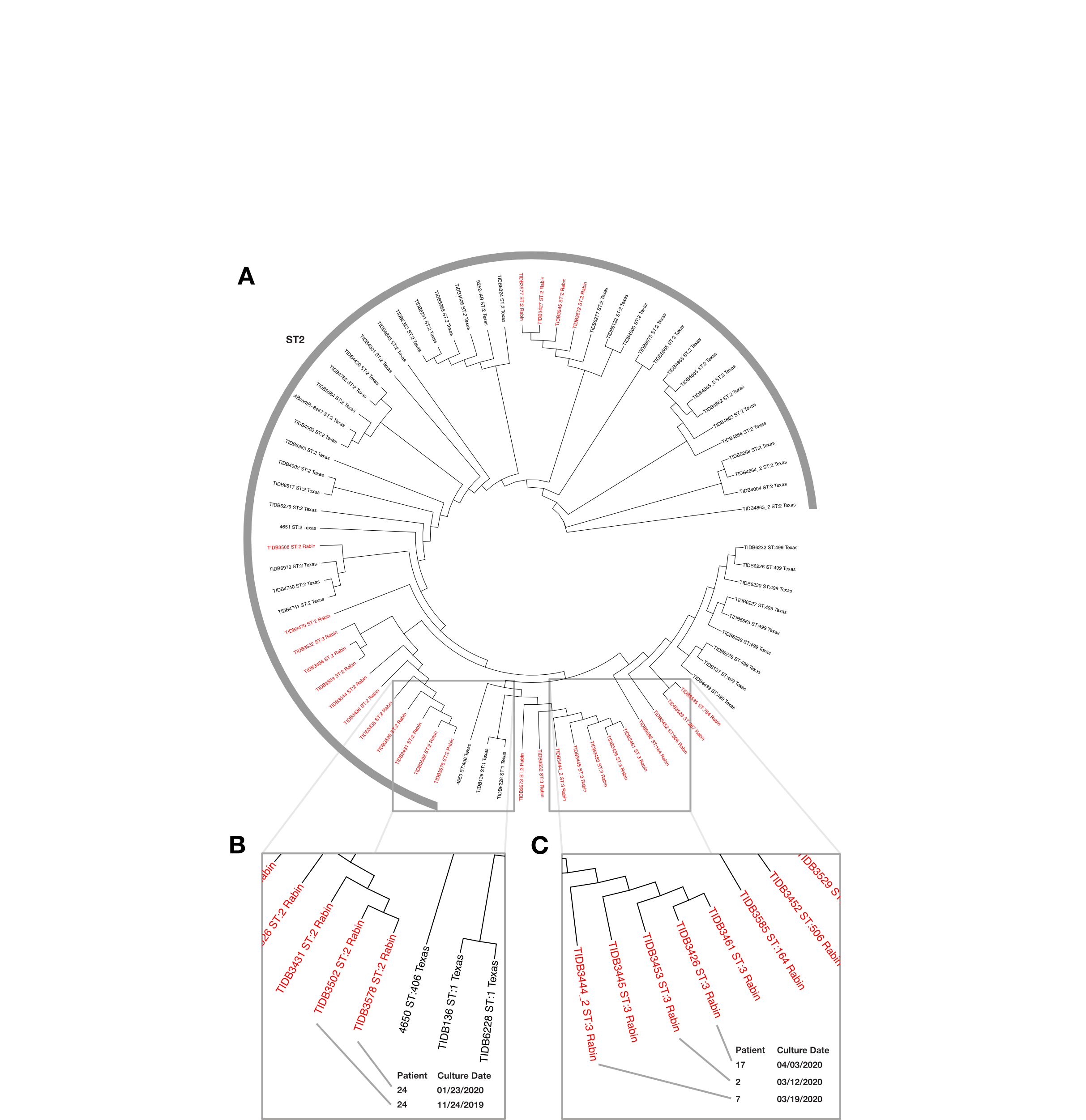
**

**(A) *A. baumannii* cladogram.** Includes isolates from Rabin Medical Center in Israel (**in red**) and UT Southwestern Medical Center in Texas (**in black**). Grey arc surrounding cladogram indicates isolates with sequence type 2, which was shared between Texas and Israel. **(B & C) Various isolates cluster within space and time.** Two directly related isolates from two hospital admissions were collected from Patient 24. Isolates from Patients 2, 7, and 17, who had overlapping hospital stays, were closely related.

**References**

1. Haenni, M., et al., *High prevalence of international ESBL CTX-M-15-producing Enterobacter cloacae ST114 clone in animals.* J Antimicrob Chemother, 2016. **71**(6): p. 1497-500.

2. Navon-Venezia, S., K. Kondratyeva, and A. Carattoli, *Klebsiella pneumoniae: a major worldwide source and shuttle for antibiotic resistance.* FEMS Microbiol Rev, 2017. **41**(3): p. 252-275.

3. David, S., et al., *Genomic surveillance of multidrug-resistant Klebsiella in Wales reveals persistent spread of Klebsiella pneumoniae ST307 and adaptive evolution of pOXA-48-like plasmids.* Microb Genom, 2023. **9**(5).

4. Ababneh, Q., et al., *Molecular epidemiology of carbapenem-resistant Acinetobacter baumannii isolated from three major hospitals in Jordan.* Int J Clin Pract, 2021. **75**(12): p. e14998.

5. McKay, S.L., et al., *Molecular Epidemiology of Carbapenem-Resistant Acinetobacter baumannii in the United States, 2013-2017.* Microb Drug Resist, 2022. **28**(6): p. 645-653.

6. Abouelfetouh, A., et al., *Diversity of carbapenem-resistant Acinetobacter baumannii and bacteriophage-mediated spread of the Oxa23 carbapenemase.* Microb Genom, 2022. **8**(2).

7. Blakiston, M.R., et al., *Epidemiology of carbapenem resistant Acinetobacter baumannii in New Zealand.* N Z Med J, 2022. **135**(1561): p. 76-82.

8. Frenk, S., et al., *Large-scale WGS of carbapenem-resistant Acinetobacter baumannii isolates reveals patterns of dissemination of ST clades associated with antibiotic resistance.* J Antimicrob Chemother, 2022. **77**(4): p. 934-943.

9. Lipworth, S., et al., *Ten-year longitudinal molecular epidemiology study of Escherichia coli and Klebsiella species bloodstream infections in Oxfordshire, UK.* Genome Med, 2021. **13**(1): p. 144.

10. Stoesser, N., et al., *Evolutionary History of the Global Emergence of the Escherichia coli Epidemic Clone ST131.* mBio, 2016. **7**(2): p. e02162.
