# Supplementary material for: Antibiotic resistance genotype, phenotype, and clinical outcomes in patients with Gram-negative infections at Rabin Medical Center in Israel
Source: Microbiol Spectr. 2024 Nov 27;13(1):e00383-24. doi: 10.1128/spectrum.00383-24 (PMC11705905; doi:10.1128/spectrum.00383-24)
Supplement: Table S1 — AST and antibiotic resistance genes present in Rabin isolates. [file spectrum.00383-24-s0002.pdf]

Sample (TIDB)	Pathogen	Sequence Type (ST)	Cefiderocol (MIC)	Meropenem (MIC)	Amikacin	Amoxicillin-clavulanate	Ampicillin	Ampicillin-Sulbactam	Aztreonam	Cefazolin	Cefotaxime	Ceftazidime	Ceftazidime-Avibactam	Ceftriaxone	Cefuroxime	Ciprofloxacin
3404	Acinetobacter_baumannii	2	0.0156	32				R				R				R
3427	Acinetobacter_baumannii	2	1	64	R			I				R		R		R
3435	Acinetobacter_baumannii	2	0.125	16	R			R				R		R		R
3470	Acinetobacter_baumannii	2	0.0625	64				R				R				R
3502	Acinetobacter_baumannii	2	0.03125	64	S			R				R		R		R
3509	Acinetobacter_baumannii	2	0.03125	32				R				R				R
3532	Acinetobacter_baumannii	2	0.03125	32				R				R				R
3545	Acinetobacter_baumannii	2	0.125	64	R			R				R		R		R
3572	Acinetobacter_baumannii	2	0.5	64												
3577	Acinetobacter_baumannii	2	0.25	64	R			S				R		R		R
3578	Acinetobacter_baumannii	2	0.125	64	S			R				R		R		R
3426	Acinetobacter_baumannii	3	0.125	32	R			R				R		R		R
3444	Acinetobacter_baumannii	3	0.125	16	R			R				R		R		R
3453	Acinetobacter_baumannii	3	0.0156	16	R			I				R		R		R
3552	Acinetobacter_baumannii	3	0.0156	16	R			S				R		R		R
3585	Acinetobacter_baumannii	164			S			S				S		I		S
3529	Acinetobacter_baumannii	267			S		R	S				I		I		S
3452	Acinetobacter_baumannii	506						S				S				S
3534	Enterobacter_cloacae	97			S	R	R	R			S	S		S	R	S
3449	Enterobacter_cloacae	107			S	R	R	R			S			S	I	S
3411	Enterobacter_cloacae	120														
3570	Enterobacter_cloacae	124														
3457	Enterobacter_cloacae	1015														
3589	Enterobacter_cloacae	1236			S	R	R	R			S	S		S	S	S
3398	Escherichia_coli	1			S	S	S	S			S	S		S	S	S
3409	Escherichia_coli	1			S	S	S	S			S	S		S	S	S
3413	Escherichia_coli	1			S	S	R	S			S	S		S		S
3416	Escherichia_coli	1			S	S	S	S			S	S		S	S	S
3428	Escherichia_coli	1			S	S	S	S			S	S		S	S	S
3516	Escherichia_coli	1			S	S	I	S			S	S		S	S	S
3397	Escherichia_coli	2			S	I	R	R			S	S		S	S	R
3393	Escherichia_coli	3			S	S	I	S			S	S		S	S	S
3418	Escherichia_coli	3			S	R	R	R			R	R		R	R	S
3430	Escherichia_coli	3			S	S	S	S			S	S		S	S	S
3454	Escherichia_coli	3			S	S	R	S			S	S		S	S	S
3547	Escherichia_coli	3			S	S	S	S			S	S		S	S	S
3555	Escherichia_coli	3			S	S	R	S			R			R	R	I
3563	Escherichia_coli	3														
3565	Escherichia_coli	3			S	I	R	R			S	S		S	S	R
3568	Escherichia_coli	3			S	R	R	R			S	S		S	R	S
3580	Escherichia_coli	3			S	S	R	S			S	S		S	S	S
3448	Escherichia_coli	4			S	S	I	S			S	S		S	S	S
3483	Escherichia_coli	4			S	S	I	S			S	S		S	S	S
3487	Escherichia_coli	4														
3499	Escherichia_coli	4			S	S	S	S			S	S		S	S	S
3571	Escherichia_coli	4			S	S	I	S			S	S		S	S	S
3576	Escherichia_coli	4			S	S	I	S			S	S		S	I	S
3441	Escherichia_coli	6			S	S	I	S			S	S		S	S	S
3447	Escherichia_coli	6														
3414	Escherichia_coli	8			S	R	R	R			R			R	R	S
3429	Escherichia_coli	8														
3513	Escherichia_coli	8			S	R	R	R			R	R		R	R	S
3582	Escherichia_coli	8														
3466	Escherichia_coli	26			S	I	R	I			R	R		R	R	I
3485	Escherichia_coli	32			S	S	R	S			S	S		S	S	S
3528	Escherichia_coli	34			S	S	I	S			S	S		S	S	S
3564	Escherichia_coli	34			S	S	S	S			S	S		S	S	S
3400	Escherichia_coli	43														
3401	Escherichia_coli	43			S	R	R	R			R	R		R	R	R
3415	Escherichia_coli	43														
3439	Escherichia_coli	43														
3446	Escherichia_coli	43			S	S	R	S			R			R	R	R
3455	Escherichia_coli	43			S	I	R	I			R			R	R	R
3459	Escherichia_coli	43			S	I	I	S			S	S		S	S	S
3460	Escherichia_coli	43			S	R	R	R			R	R		R	R	R
3464	Escherichia_coli	43			I	R	R	R			R			R	R	R
3478	Escherichia_coli	43			S	R	R	R			R			R	R	R
3488	Escherichia_coli	43			S	I	R	I			R	R		R	R	R
3504	Escherichia_coli	43			S	R	R	R			R	R		R	R	R
3511	Escherichia_coli	43			S	I	R	R			R	R		R	R	R
3514	Escherichia_coli	43			S	R		R			R	R		R	R	R
3540	Escherichia_coli	43														
3551	Escherichia_coli	43			S	I	R				S	S		S	S	
3556	Escherichia_coli	43			S	R	R	R			R	R		R	R	R
3569	Escherichia_coli	43			S	R	R	R			R	R		R	R	R
3581	Escherichia_coli	43														
3523	Escherichia_coli	48			S	S	I	S			S	S		S	S	S
3399	Escherichia_coli	52			S	I	R	R			S	S		S	S	S
3424	Escherichia_coli	52			S	S	S	S			S	S		S	S	S
3425	Escherichia_coli	52			S	S	R				S	S		S	S	S
3530	Escherichia_coli	53			S	S	R	S			S	S		S	S	R
3479	Escherichia_coli	55			S	S	R	S	S		S	S		S	S	S
3391	Escherichia_coli	70			S	S	I	S			S	S		S	S	S
3567	Escherichia_coli	83			S	R	R	R			R	R		R	R	I
3395	Escherichia_coli	129														
3482	Escherichia_coli	404			S	S	S	S			S	S		S	S	S
3507	Escherichia_coli	455			S	I	R	S			S	S		S	S	S
3410	Escherichia_coli	478														
3436	Escherichia_coli	478														
3437	Escherichia_coli	478			S	S	S	S			S	S		S	S	S
3412	Escherichia_coli	506														
3587	Escherichia_coli	506			S	R	R	R			R			R	R	R
3558	Escherichia_coli	511			S	I	R	S			S	S		S	S	S
3575	Escherichia_coli	535			S	S	R	S			S	S		S	S	S
3417	Escherichia_coli	638			S	S	R	S			S	S		S	S	S
3503	Escherichia_coli	650			S	I	R	R			S	S		S	I	R
3442	Escherichia_coli	730			S	S	I	S			S	S		S	S	S
3586	Escherichia_coli	803														
3468	Escherichia_coli	940			S	S	S	S			S	S		S	S	S
3583	Escherichia_coli	956			S	R	R	R			S	S		S	R	S
3484	Escherichia_coli	972			S	S	I	S			S	S		S	S	S
3566	Escherichia_coli	678_722			S	S	I	S			S	S		S	S	S
3419	Klebsiella_pneumoniae	17			S	S	R	S			S	S		S	S	S
3422	Klebsiella_pneumoniae	23														
3477	Klebsiella_pneumoniae	23			S	S	R	I			S	S		S	S	S
3549	Klebsiella_pneumoniae	29			S	I	R	S			R			R	R	I
3525	Klebsiella_pneumoniae	35			S	I	R	I			R			R	R	R
3458	Klebsiella_pneumoniae	45			S	I	R	R			S	S		S	R	I
3465	Klebsiella_pneumoniae	45			S	R	R	R			S	S		S	S	S
3517	Klebsiella_pneumoniae	101			I	R	R	R			R	R		R	R	R
3434	Klebsiella_pneumoniae	225			I	R	R	R			R	R		R	R	I
3548	Klebsiella_pneumoniae	252														
3392	Klebsiella_pneumoniae	307			S	I	R	I			R			R	R	R
3560	Klebsiella_pneumoniae	405			S	R	R	R			R	R		R	R	S
3574	Klebsiella_pneumoniae	636			S	S	R	S			S	S		S	S	S
3407	Klebsiella_pneumoniae	716			R	R	R	R			R	R	S	R	R	R
3550	Klebsiella_pneumoniae	785														
3561	Klebsiella_pneumoniae	927			S	S	R	S			S	S		S	S	R
3433	Klebsiella_pneumoniae	1303			S	S	R				S	S		S	S	S
3510	Klebsiella_pneumoniae	1563			S	R	R	R			S	I		S	I	S
3498	Klebsiella_pneumoniae	1666			S	S	R	S			S	S		S	S	S
3542	Klebsiella_pneumoniae	1779			S	R	R	R			R	R		R	R	I
3515	Klebsiella_pneumoniae	1825														
3463	Klebsiella_pneumoniae	2407			S	I	R	I			R	R		R	R	I
3481	Klebsiella_pneumoniae	4712			S	S	R	I			S	S		S	S	I
3541	Klebsiella_pneumoniae	1516_1873			S	S	R	S			S	S		S	S	S
3531	Pseudomonas_aeruginosa	111	0.5		R							R	R			R
3536	Pseudomonas_aeruginosa	111			S							S				R
3443	Pseudomonas_aeruginosa	137			S				S			S				S
3505	Pseudomonas_aeruginosa	233			R							R				R
3402	Pseudomonas_aeruginosa	235			S				S			S	S			S
3518	Pseudomonas_aeruginosa	235	0.03125		S							I				R
3521	Pseudomonas_aeruginosa	235			S							S				S
3522	Pseudomonas_aeruginosa	244	0.25		I				R			S	S			R
3445	Pseudomonas_aeruginosa	253			S				S			S				S
3451	Pseudomonas_aeruginosa	253	0.03125		S				S			S	S			R
3475	Pseudomonas_aeruginosa	253			S							S				S
3524	Pseudomonas_aeruginosa	253			S							S				S
3462	Pseudomonas_aeruginosa	270			S				S			S	S			S
3471	Pseudomonas_aeruginosa	274			S							S				S
3495	Pseudomonas_aeruginosa	293	0.25		R							R				R
3554	Pseudomonas_aeruginosa	293	0.25		S							S				R
3539	Pseudomonas_aeruginosa	316	0.125		S							R				S
3584	Pseudomonas_aeruginosa	319	0.125		S							S				S
3473	Pseudomonas_aeruginosa	395			S							S				S
3494	Pseudomonas_aeruginosa	408			S	R	R			R	R	S		R	R	S
3543	Pseudomonas_aeruginosa	532			S							S				R
3588	Pseudomonas_aeruginosa	532	0.125		S							S	S			R
3450	Pseudomonas_aeruginosa	545			S				S			S	S			R
3496	Pseudomonas_aeruginosa	557			S							S				S
3489	Pseudomonas_aeruginosa	644			S				R			S	S			R
3491	Pseudomonas_aeruginosa	644														
3497	Pseudomonas_aeruginosa	644	0.25		S				R			R	S			S
3519	Pseudomonas_aeruginosa	644			S							R				S
3538	Pseudomonas_aeruginosa	644	0.125		S				R			R	S			S
3486	Pseudomonas_aeruginosa	654	0.5		S				I			R	S			R
3438	Pseudomonas_aeruginosa	834			S				S			S				S
3474	Pseudomonas_aeruginosa	1007			S							S				S
3480	Pseudomonas_aeruginosa	1194			S							S				S
3559	Pseudomonas_aeruginosa	1284	0.25		S							S				S
3467	Pseudomonas_aeruginosa	1600			S				S			S	S			S
3396	Pseudomonas_aeruginosa	1800			S				S			S				S
3456	Pseudomonas_aeruginosa	2049			S				S			S				S
3562	Pseudomonas_aeruginosa	2068			S							S				S
3553	Pseudomonas_aeruginosa	2326														
3469	Pseudomonas_aeruginosa	3069			S				S			S	S			S
3520	Pseudomonas_aeruginosa	3223	0.0625		S							R				R
3490	Pseudomonas_aeruginosa	1047_2608	0.25		S				I			S	S			S

Sample (TIDB)	Colistin	Ertapenem	Fosfomycin	Gentamicin	Imipenem	Meropenem	Minocycline	Ofloxacin	Piperacillin	Piperacillin-Tazobactam	Tigecycline	Trimethoprim-Sulfamethoxazole	AAC(3)-Ia	AAC(3)-Ib	AAC(3)-IIa	AAC(3)-IIc	AAC(3)-IVa	AAC(6')-29a	AAC(6')-Iaf	AAC(6')-Ib10	AAC(6')-Ib9	AAC(6')-Ib-cr
3404				R	R	R	S			R	R	R	0	0	0	0	0	0	0	0	0	0
3427	S			R	R	R	I			R	R	R	1	1	0	0	0	0	1	0	0	0
3435	S			R	R	R	I			R	R	S	0	0	0	0	0	0	0	0	0	0
3470	S		R	R	R	R	I			R	R	S	1	1	0	0	0	0	1	0	0	0
3502	S			I	R	R	I			R	R	I	0	0	0	0	0	0	0	0	0	0
3509	S			R	R	R	R			R	R	R	0	0	0	0	0	0	0	0	0	0
3532	S		R	R	R	R	R			R	R	R	0	0	0	0	0	0	0	0	0	0
3545	S			R	R	R	R			R	R	R	1	1	0	0	0	0	1	0	0	0
3572						R							1	1	0	0	0	0	1	0	0	0
3577	S			R	R	R	R			R	R	R	1	1	0	0	0	0	1	0	0	0
3578	S			I	R	R	I			R	R	I	0	0	0	0	0	0	0	0	0	0
3426	S			R	R	R	S			R	R	R	0	0	0	0	0	0	0	0	0	0
3444	S			R	R	R	R			R	R	R	0	0	0	0	0	0	0	0	0	0
3453	S			R	R	R	S			R	R	R	0	0	0	0	0	0	0	0	0	0
3552	S			R	R	R	S			R	R	R	0	0	0	0	0	0	0	0	0	0
3585				S	S	S	S			S		S	0	0	0	0	0	0	0	0	0	0
3529				S	S	S	S			I		S	0	0	0	0	0	0	0	0	0	0
3452	S		R	S	S	S	S			S	S	S	0	0	0	0	0	0	0	0	0	0
3534		S		S	S	S	I	S	S	S		S	0	0	0	0	0	0	0	0	0	0
3449		S		S	S	S		S	S	S		S	0	0	0	0	0	0	0	0	0	0
3411													0	0	0	0	0	0	0	0	0	0
3570													0	0	0	0	0	0	0	0	0	0
3457													0	0	0	0	0	0	0	0	0	0
3589		S		S	S	S	R	S	S	S		S	0	0	0	0	0	0	0	0	0	0
3398		S		S	S	S		S	S	S		S	0	0	0	0	0	0	0	0	0	0
3409		S		S	S	S		S	S	S		S	0	0	0	0	0	0	0	0	0	0
3413		S		S	S	S		S	R	S		S	0	0	0	0	0	0	0	0	0	0
3416		S		S	S	S		S	S	S		S	0	0	0	0	0	0	0	0	0	0
3428		S		S	S	S		S	S	S		S	0	0	0	0	0	0	0	0	0	0
3516		S		S	S	S	R	S	S	S		S	0	0	0	0	0	0	0	0	0	0
3397		S		R	S	S		R	R	S		R	0	0	1	0	0	0	0	0	0	0
3393		S		S	S	S		S	S	S		S	0	0	0	0	0	0	0	0	0	0
3418		S		S	S	S		S	R	R		R	0	0	0	0	0	0	0	0	0	0
3430		S		S	S	S		S	S	S		S	0	0	0	0	0	0	0	0	0	0
3454		S		S	S	S		S	R	S		S	0	0	0	0	0	0	0	0	0	0
3547		S		S	S	S		S	S	S		S	0	0	0	0	0	0	0	0	0	0
3555		S		S	S	S	R	S	R	S		R	0	0	0	0	0	0	0	0	0	0
3563													0	0	0	0	0	0	0	0	0	0
3565		S		S	S	S	S	R	R	S		R	0	0	0	0	0	0	0	0	0	0
3568		S		S	S	S	S	S		S		R	0	0	0	0	0	0	0	0	0	0
3580		S		R	S	S	S	S	R	S		R	0	0	1	0	0	0	0	0	0	0
3448		S		S	S	S		S	S	S		S	0	0	0	0	0	0	0	0	0	0
3483		S		S	S	S	S	S	S	S		S	0	0	0	0	0	0	0	0	0	0
3487													0	0	0	0	0	0	0	0	0	0
3499		S		S	S	S	S	S	S	S		S	0	0	0	0	0	0	0	0	0	0
3571		S		S	S	S	S	S	S	S		S	0	0	0	0	0	0	0	0	0	0
3576		S		S	S	S	S	S	S	S		S	0	0	0	0	0	0	0	0	0	0
3441		S		S	S	S		S	S	S		S	0	0	0	0	0	0	0	0	0	0
3447													0	0	0	0	0	0	0	0	0	0
3414		S		S	S	S		S	R	S		R	0	0	0	0	0	0	0	0	0	0
3429													0	0	0	1	0	0	0	1	0	0
3513		S		S	S	S	I	S	R	S		R	0	0	0	0	0	0	0	0	0	0
3582													0	0	0	0	0	0	0	0	0	0
3466		S		S	S	S	S	S	R	S		S	0	0	0	0	0	0	0	0	0	0
3485		S		S	S	S	R	S	R	S		S	0	0	0	0	0	0	0	0	0	0
3528		S		S	S	S	S	S	S	S		S	0	0	0	0	0	0	0	0	0	0
3564		S		S	S	S	S	S	S	S		S	0	0	0	0	0	0	0	0	0	0
3400													0	0	0	0	0	0	0	0	0	0
3401		S		R	S	S	I	R	R	S		R	0	0	0	1	0	0	0	0	0	1
3415													0	0	1	0	0	0	0	0	0	1
3439													0	0	0	0	0	0	0	0	0	0
3446		S		S	S	S		R	R	S		R	0	0	0	0	0	0	0	0	0	0
3455		S		S	S	S		R	R	S		R	0	0	0	0	0	0	0	0	0	0
3459		S		S	S	S		S	S	S		S	0	0	0	0	0	0	0	0	0	0
3460		S		S	S	S	S	R	R	S		S	0	0	0	0	0	0	0	0	0	0
3464		S		S	S	S		R	R	I		R	0	0	0	0	0	0	0	0	0	1
3478		S		R	S	S		R	R	S		S	0	0	0	1	0	0	0	0	0	1
3488		S		S	S	S	S	R	R	I		R	0	0	0	0	0	0	0	0	0	0
3504		S		R	S	S	I	R	R	I		R	0	0	1	0	0	0	0	0	0	1
3511		S		R	S	S	S	R	R	S		R	0	0	0	1	0	0	0	0	0	1
3514		S		S	S	S	S	R	R	S		R	0	0	0	0	0	0	0	0	0	1
3540													0	0	0	0	0	0	0	0	0	0
3551		S		R	S	S	S	R	S	S		R	0	0	0	1	0	0	0	0	0	1
3556		S		S	S	S	S	R	R	I		R	0	0	0	0	0	0	0	0	0	1
3569		S		S	S	S	S	R	R	S		S	0	0	0	0	0	0	0	0	0	1
3581													0	0	0	0	0	0	0	0	0	1
3523		S		S	S	S	S	S	S	S		S	0	0	0	0	0	0	0	0	0	0
3399		S		S	S	S		S	R	S		S	0	0	0	0	0	0	0	0	0	0
3424		S		S	S	S		S	S	S		S	0	0	0	0	0	0	0	0	0	0
3425		S		S	S	S		S	R	S		S	0	0	0	0	0	0	0	0	0	0
3530		S		R	S	S	S	R	R	S		R	0	0	1	0	0	0	0	0	0	0
3479		S		S	S	S		S	S	S		S	0	0	0	0	0	0	0	0	0	0
3391		S		S	S	S		S	S	S		S	0	0	0	0	0	0	0	0	0	0
3567		S		R	S	S	S	S	R	S		R	0	0	0	0	0	0	0	0	0	0
3395													0	0	0	0	0	0	0	0	0	0
3482		S		S	S	S	S	S	S	S		S	0	0	0	0	0	0	0	0	0	0
3507		S		S	S	S	S	S	R	S		R	0	0	0	0	0	0	0	0	0	0
3410													0	0	0	0	0	0	0	0	0	0
3436													0	0	1	0	0	0	0	0	0	0
3437		S		S	S	S		S	S	S		S	0	0	1	0	0	0	0	0	0	0
3412													0	0	0	0	0	0	0	0	0	0
3587		S		S	S	S	S	S	R	I		S	0	0	0	0	0	0	0	0	0	0
3558		S		S	S	S	S	S	S	S		S	0	0	0	0	0	0	0	0	0	0
3575		S		S	S	S	S	S	S	S		S	0	0	0	0	0	0	0	0	0	0
3417		S		S	S	S		S	R	S		R	0	0	0	0	0	0	0	0	0	0
3503		S		R	S	S	I	R	R	S		R	0	0	1	0	0	0	0	0	0	0
3442		S		S	S	S		S	S	S		S	0	0	0	0	0	0	0	0	0	0
3586													0	0	0	0	0	0	0	0	0	0
3468		S		S	S	S	S	S	S	S		S	0	0	0	0	0	0	0	0	0	0
3583		S		S	S	S	S	S		I		S	0	0	0	0	0	0	0	0	0	0
3484		S		S	S	S	R	S	S	S		S	0	0	0	0	0	0	0	0	0	0
3566		S		S	S	S	S	S	S	S		S	0	0	0	0	0	0	0	0	0	0
3419		S		S	S	S		S	S	S		S	0	0	0	0	0	0	0	0	0	0
3422													0	0	0	0	0	0	0	0	0	0
3477		S		S	S	S		S	I	S		S	0	0	0	0	0	0	0	0	0	0
3549		S		S	S	S	S	S	R	I		R	0	0	0	0	0	0	0	0	0	0
3525		S		S	S	S	S	S	R	S		R	0	0	0	0	0	0	0	0	0	1
3458		S		S	S	S		S	I	I		S	0	0	0	0	0	0	0	0	0	0
3465		S		S	S	S		S	R	S		S	0	0	0	0	0	0	0	0	0	0
3517		S		R	S	S	S	R	R	I		R	0	0	0	1	0	0	0	0	0	0
3434		S		R	S	S		S	R	I		S	0	0	0	1	0	0	0	1	0	0
3548													0	0	0	0	0	0	0	0	0	0
3392		S		S	S	S		I	R	S		R	0	0	0	0	0	0	0	0	0	1
3560		S		R	S	S	S	S	R	S		R	0	0	0	1	0	0	0	0	0	1
3574		S		S	S	S	S	S	S	S		S	0	0	0	0	0	0	0	0	0	0
3407		S		R	S	S		S	R	R		R	0	0	0	1	0	0	0	0	0	1
3550													0	0	0	0	0	0	0	0	0	0
3561		S		R	S	S	S	R	S	S		R	0	0	1	0	1	0	0	0	0	0
3433		S		S	S	S		S	S	S		S	0	0	0	0	0	0	0	0	0	0
3510		S		S	S	S	R	S	R	R		I	0	0	0	0	0	0	0	0	0	0
3498		S		S	S	S	S	S	S	S		S	0	0	0	0	0	0	0	0	0	0
3542		S		S	S	S	R	S	R	S		R	0	0	0	0	0	0	0	0	0	0
3515													0	0	0	0	0	0	0	0	0	0
3463		S		R	S	S		S	R	S		R	0	0	1	0	0	0	0	0	0	0
3481		S		S	S	S	S	S	I	S		R	0	0	0	0	0	0	0	0	0	0
3541		S		S	S	S	S	S	S	S		I	0	0	0	0	0	0	0	0	0	0
3531	S		S	I	R	I			I	I			0	0	0	0	0	1	0	0	0	0
3536				R	S	S			R	I			0	0	0	0	0	0	0	0	1	0
3443				S	S	S			S	S			0	0	0	0	0	0	0	0	0	0
3505				R	S	S			R	R			0	0	0	0	0	0	0	0	0	0
3402				S	S	S			S	S			0	0	0	0	0	0	0	0	0	0
3518	S		S	R	R	R			R	R			0	0	0	0	0	0	0	0	0	0
3521				S	S	S			S	S			0	0	0	0	0	0	0	0	0	0
3522	S		R	R	R	R			R	R			0	0	0	0	0	0	0	0	1	0
3445				S	S	S			S	S			0	0	0	0	0	0	0	0	0	0
3451				S	R	R			S	S			0	0	0	0	0	0	0	0	0	0
3475				S	S	S			S	S			0	0	0	0	0	0	0	0	0	0
3524				S	S	S			S	S			0	0	0	0	0	0	0	0	0	0
3462				S	S	S			S	S			0	0	0	0	0	0	0	0	0	0
3471				S	S	S			R	S			0	0	0	0	0	0	0	0	0	0
3495	S		R	R	R	R			R	R			0	0	0	0	0	0	0	0	0	0
3554	S		R	S	R	R			I	I			0	0	0	0	0	0	0	0	0	0
3539	S		R	S	R	R			R	R			0	0	0	0	0	0	0	0	0	0
3584				S	R	R			S	S			0	0	0	0	0	0	0	0	0	0
3473				S	S	S			S	S			0	0	0	0	0	0	0	0	0	0
3494		S		S	S	S	S		S	S		R	0	0	0	0	0	0	0	0	0	0
3543				I	S	S			S	S			0	0	0	0	0	0	0	0	0	0
3588	R			S	R	R			I	I			0	0	0	0	0	0	0	0	0	0
3450				S	S	S			S	S			0	0	0	0	0	0	0	0	0	0
3496				S	S	S			S	S			0	0	0	0	0	0	0	0	0	0
3489				S	S	S			I	S			0	0	0	0	0	0	0	0	0	0
3491													0	0	0	0	0	0	0	0	0	0
3497	S		R	S	R	R			R	R			0	0	0	0	0	0	0	0	0	0
3519				S	S	S			R	R			0	0	0	0	0	0	0	0	0	0
3538	S		R	S	R	R			R	R			0	0	0	0	0	0	0	0	0	0
3486	S			R	R	R			I	I			0	0	0	0	0	0	0	0	1	0
3438				S	S	S			S	S			0	0	0	0	0	0	0	0	0	0
3474				S	S	S			S	S			0	0	0	0	0	0	0	0	0	0
3480				S	S	S			S	S			0	0	0	0	0	0	0	0	0	0
3559	S		S	R	R	I			S	S			0	0	0	0	0	0	0	0	0	0
3467				S	S	S			S	S			0	0	0	0	0	0	0	0	0	0
3396				I	S	S			S	S			0	0	0	0	0	0	0	0	0	0
3456				S	S	S			S	S			0	0	0	0	0	0	0	0	0	0
3562				S	S	S			S	S			0	0	0	0	0	0	0	0	0	0
3553													0	0	0	0	0	0	0	0	0	0
3469				S	S	S			S	S			0	0	0	0	0	0	0	0	0	0
3520	S		R	S	R	I			R	R			0	0	0	0	0	0	0	0	0	0
3490	S		R	S	R	R			R	R			0	0	0	0	0	0	0	0	0	0

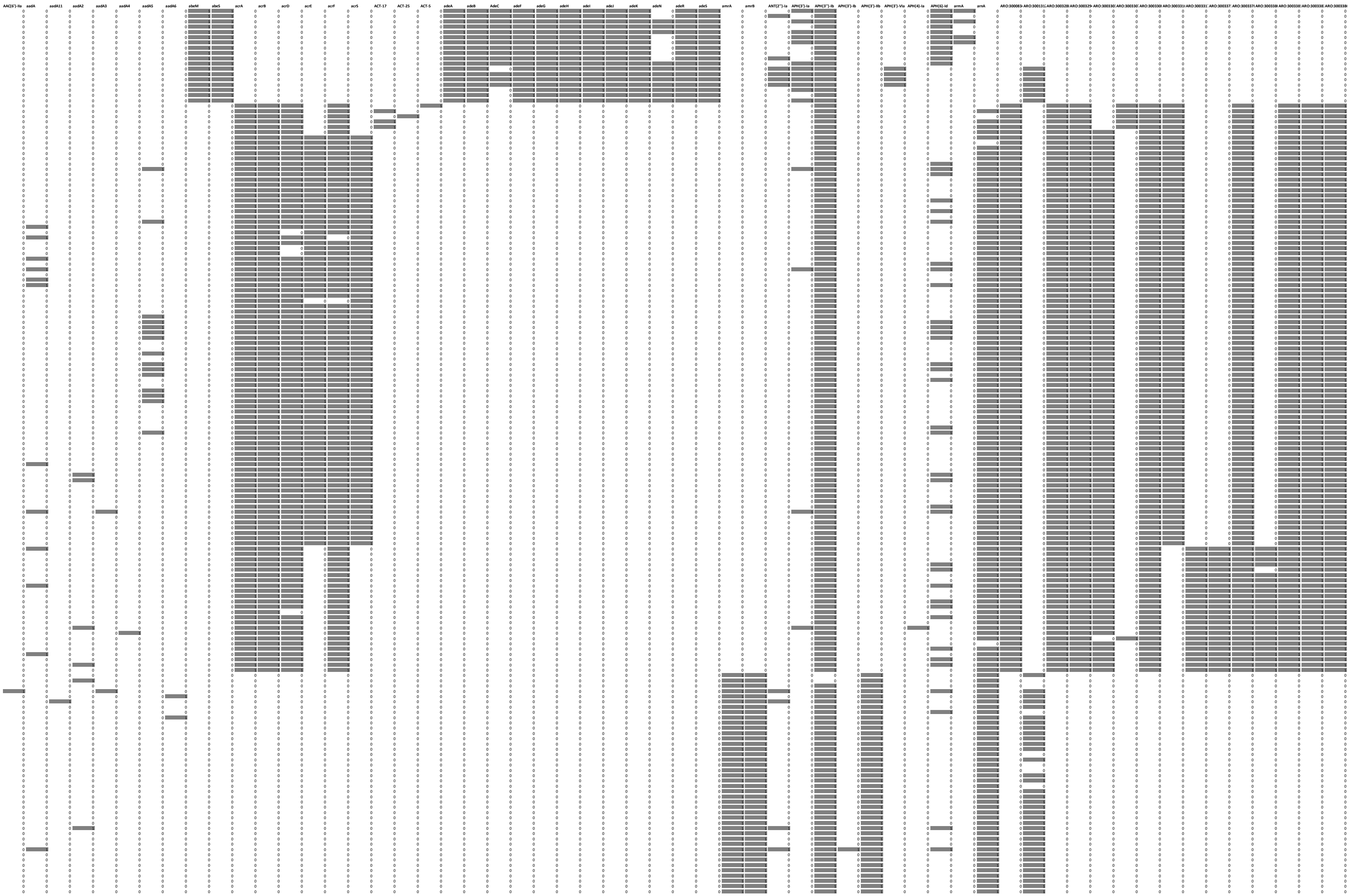

AAC(6')-IIa
aadA
aadA11
aadA2
aadA3
aadA4
aadA5
aadA6
abeM
abeS
acrA
acrB
acrD
acrE
acrF
acrS
ACT-17
ACT-25
ACT-5
adeA
adeB
AdeC
adeF
adeG
adeH
adeI
adeJ
adeK
adeN
adeR
adeS
amrA
amrB
ANT(2'')-Ia
APH(3')-Ia
APH(3'')-Ib
APH(3')-Ib
APH(3')-IIb
APH(3')-VIa
APH(4)-Ia
APH(6)-Id
armA
arnA

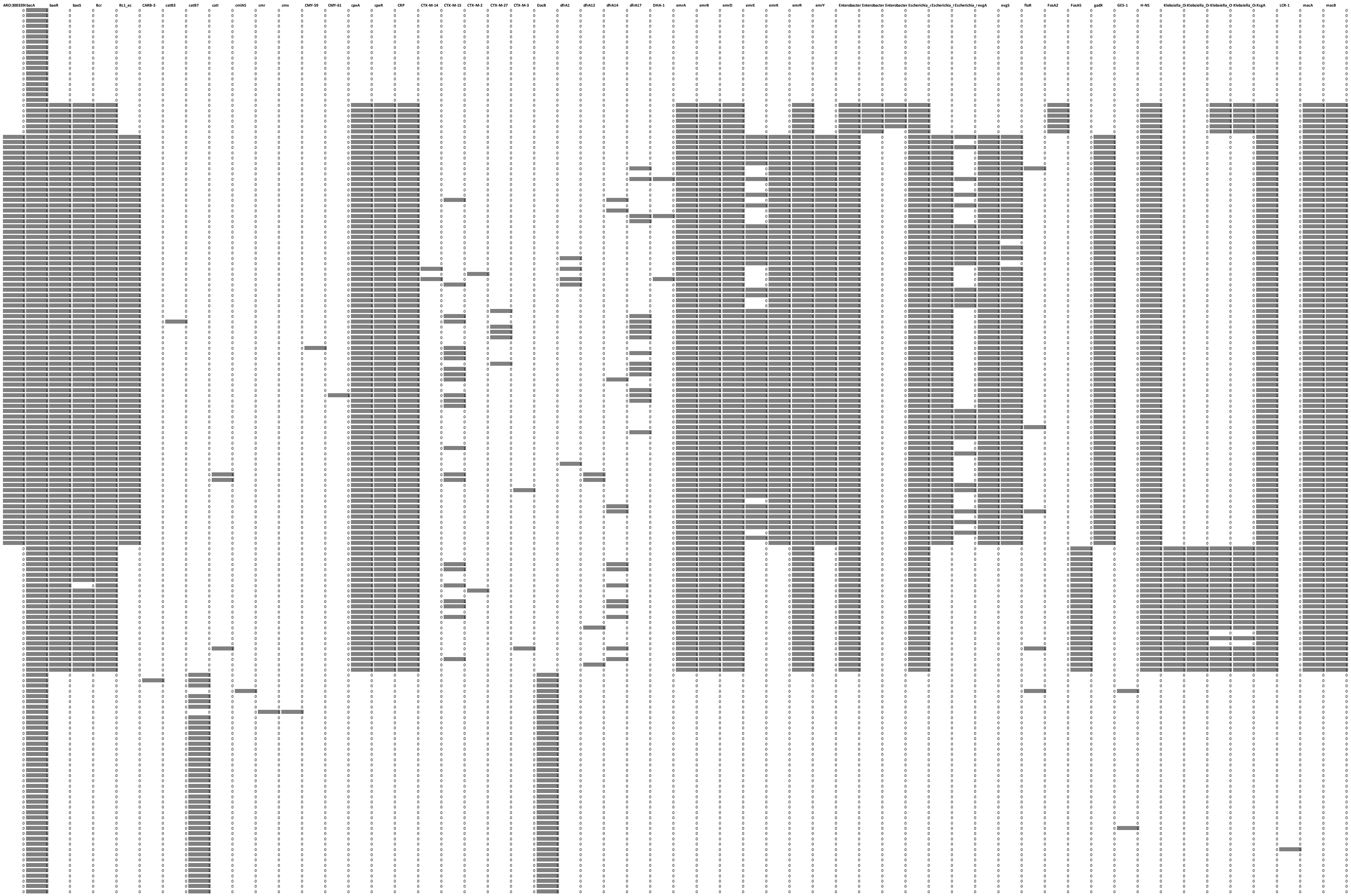

ARO:300339(
bacA
baeR
baeS
Bcr
BL1_ec
CARB-3
catB3
catB7
catI
cmlA5
cmr
cmx
CMY-59
CMY-61
cpxA
cpxR
CRP
CTX-M-14
CTX-M-15
CTX-M-2
CTX-M-27
CTX-M-3
DacB
dfrA1
dfrA12
dfrA14
dfrA17
DHA-1
emrA
emrB
emrD
emrE
emrK
emrR
emrY
Enterobacter
Enterobacter
Enterobacter
Escherichia_
Escherichia_
Escherichia_
evgA
evgS
floR
FosA2
FosA5
gadX
GES-1
H-NS
Klebsiella_O
Klebsiella_O
Klebsiella_O
Klebsiella_O
KsgA
LCR-1
macA
macB

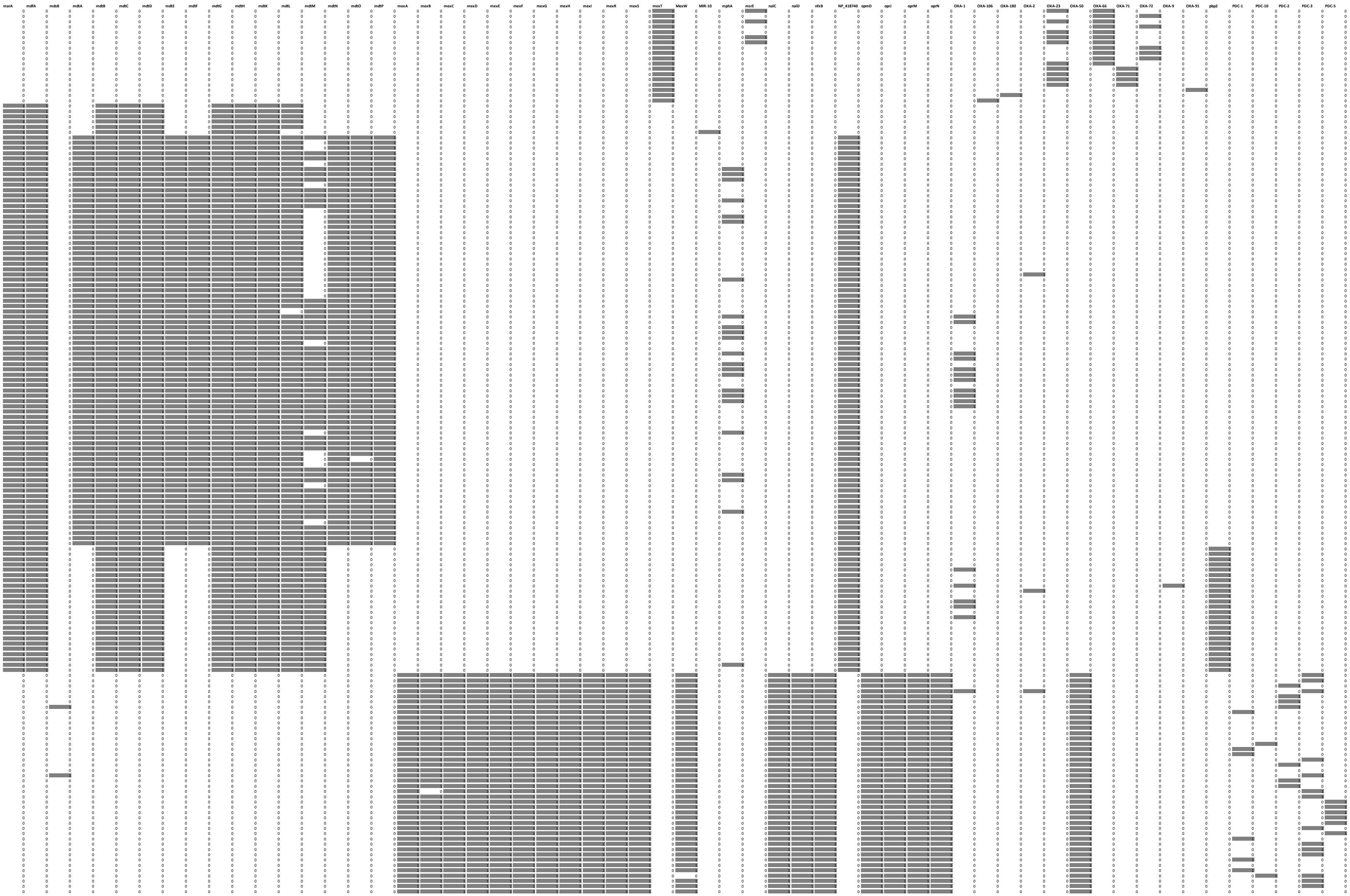
marA
mdfA
mdsB
mdtA
mdtB
mdtC
mdtD
mdtE
mdtF
mdtG
mdtH
mdtK
mdtL
mdtM
mdtN
mdtO
mdtP
mexA
mexB
mexC
mexD
mexE
mexF
mexG
mexH
mexI
mexR
mexS
mexT
MexW
MIR-10
mphA
msrE
nalC
nalD
nfxB
NP_418740
opmD
oprJ
oprM
oprN
OXA-1
OXA-106
OXA-180
OXA-2
OXA-23
OXA-50
OXA-66
OXA-71
OXA-72
OXA-9
OXA-91
pbp2
PDC-1
PDC-10
PDC-2
PDC-3
PDC-5

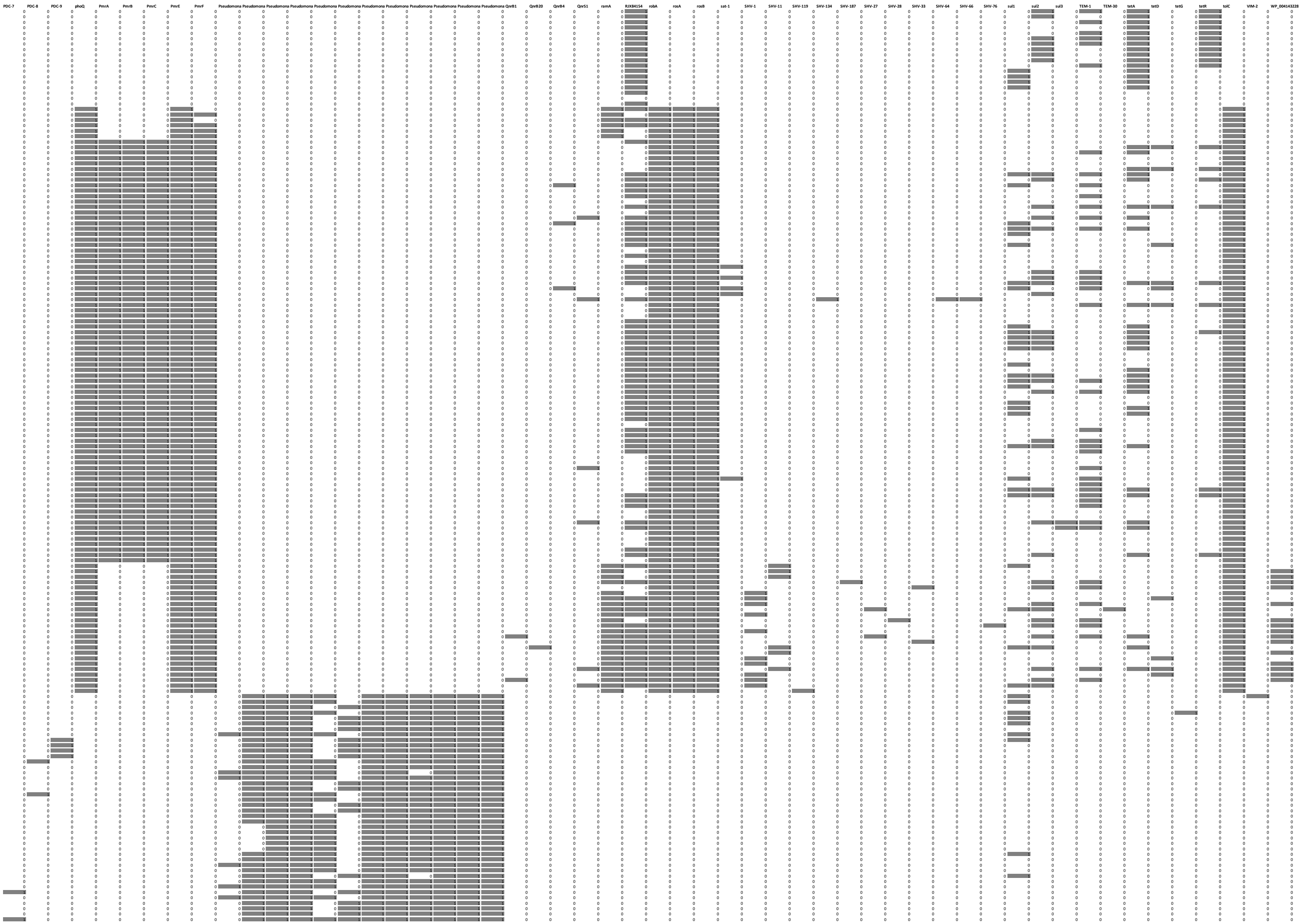
PDC-7
PDC-8
PDC-9
phoQ
PmrA
PmrB
PmrC
PmrE
PmrF
Pseudomona
Pseudomona
Pseudomona
Pseudomona
Pseudomona
Pseudomona
Pseudomona
Pseudomona
Pseudomona
Pseudomona
Pseudomona
Pseudomona
QnrB1
QnrB20
QnrB4
QnrS1
ramA
RJX84154
robA
rosA
rosB
sat-1
SHV-1
SHV-11
SHV-119
SHV-134
SHV-187
SHV-27
SHV-28
SHV-33
SHV-64
SHV-66
SHV-76
sul1
sul2
sul3
TEM-1
TEM-30
tetA
tetD
tetG
tetR
tolC
VIM-2
WP_004143228
